# On Bayes factors for hypothesis tests

**DOI:** 10.3758/s13423-024-02612-2

**Published:** 2024-11-25

**Authors:** Karl Christoph Klauer, Constantin G. Meyer-Grant, David Kellen

**Affiliations:** 1 Department of Psychology, Albert-Ludwigs-Universität Freiburg, 79085 Freiburg, Germany; 2 Department of Psychology, Syracuse University, Syracuse, NY USA

**Keywords:** Bayesian statistics, Statistical inference, Default Bayes factors

## Abstract

We develop alternative families of Bayes factors for use in hypothesis tests as alternatives to the popular default Bayes factors. The alternative Bayes factors are derived for the statistical analyses most commonly used in psychological research – one-sample and two-sample *t* tests, regression, and ANOVA analyses. They possess the same desirable theoretical and practical properties as the default Bayes factors and satisfy additional theoretical desiderata while mitigating against two features of the default priors that we consider implausible. They can be conveniently computed via an R package that we provide. Furthermore, hypothesis tests based on Bayes factors and those based on significance tests are juxtaposed. This discussion leads to the insight that default Bayes factors as well as the alternative Bayes factors are equivalent to test-statistic-based Bayes factors as proposed by Johnson. *Journal of the Royal Statistical Society Series B: Statistical Methodology*, *67*, 689–701. (2005). We highlight test-statistic-based Bayes factors as a general approach to Bayes-factor computation that is applicable to many hypothesis-testing problems for which an effect-size measure has been proposed and for which test power can be computed.

**Supplementary Information:**

The online version contains supplementary material available at 10.3758/s13423-024-02612-2.

Recent years have seen a notable increase in the use of Bayesian statistical methods for the purpose of hypothesis testing, replacing established frequentist solutions (e.g., McElreath, 2020; Wagenmakers et al., 2018). In the vast majority of cases, this transition has taken the form of researchers opting for *Bayes factors* as their instrument of hypothesis evaluation, rather than adhering to the traditional metric of frequentist *p* values (Heck et al., 2023).

Bayes factors quantify the evidence for an alternative hypothesis relative to a null hypothesis (and conversely), when said hypotheses are represented by probabilistic models. For a given pair of competing models $\mathcal {M}_1$ and $\mathcal {M}_0$ and data ${\varvec{y}}$, the Bayes factor is defined as

1 $$\begin{aligned} B_{10} = \frac{l({\varvec{y}} \mid \mathcal {M}_1)}{l({\varvec{y}} \mid \mathcal {M}_0)}, \end{aligned}$$

where $l(\varvec{y} \mid \mathcal {M})$ is a model’s marginal likelihood.

One of the attractive features of Bayes factors that have made them so popular recently is their straightforward interpretation: How much more likely are the data under model $\mathcal {M}_1$ (i.e., were $\mathcal {M}_1$ true) compared to model $\mathcal {M}_0$ (i.e., were $\mathcal {M}_0$ true). For example, “a Bayes factor $B_{10} = 10$ means that the data are ten times more probable under $\mathcal {M}_1$ than under $\mathcal {M}_0$” (Rouder & Morey, 2012, p. 881). A more technical definition of Bayes factors is provided in Table 1.

This popularity is also partly due to the existing suite of data-analytic solutions implementing their computation for standard statistical methods such as *t* tests, regression, ANOVA, and sequential testing (e.g., Morey & Rouder, 2013; Schönbrodt et al., 2017; Wagenmakers et al., 2018; Wetzels et al., 2012). Many of these solutions rely on a special class of priors, which includes the so-called Jeffreys-Zellner-Siow (JZS) priors (e.g., Liang et al., 2008, Rouder et al., 2009), that afford enormous simplifications of the integration problem implied by the computation of Bayes factors (see Table 1). Adopting these priors typically reduces the integration from a high-dimensional one to an integration over just one variable (but see Rouder et al., 2012), which can be performed relatively efficiently. Rouder, Morey, and colleagues call these priors “default priors” (Rouder et al., 2009, 2012; Rouder & Morey, 2012). Integration problems aside, these data-analytic solutions also attempt to strike a balance between two contrasting needs: On one hand, a demand for general, user-friendly tools that can be easily adopted by a wide range of researchers and practitioners, including those with limited technical expertise. On the other, a desire for researchers and practitioners to be more closely involved in shaping the statistical models, integrating their own prior beliefs into the analysis.

**Table 1:** Notation and terminology

Symbol	Definitions
$\varvec{y}$	The *data* $y_1$, $y_2$, $\ldots$, $y_N$ are collected in a vector ${\varvec{y}} = (y_1, \ldots , y_N)^t$.
$\varvec{\theta }$	*Parameters* will be denoted by Greek letters such as ${\varvec{\theta }} = (\theta _1,\ldots , \theta _k)^t$.
$f({\varvec{y}} \mid {\varvec{\theta }})$	The data are distributed according to a parameterized family of densities (or mass functions) $f({\varvec{y}} \mid {\varvec{\theta }})$, defining the *data likelihood*.
$f({\varvec{\theta }})$	A *prior* is a distribution defined over the parameter space with density (or mass function) $f({\varvec{\theta }})$.
$\mathcal {M}$	In Bayesian statistics, a *model* $\mathcal {M}$ is defined by likelihood *and* prior.
$l({\varvec{y}} \mid \mathcal {M})$	A model’s *marginal likelihood* is defined as
	$$l({\varvec{y}} \mid \mathcal {M}) = \int f({\varvec{y}} \mid {\varvec{\theta }}) f({\varvec{\theta }}) \text {d}{\varvec{\theta }}$$.
$B_{10}$	The *Bayes factor* for model $\mathcal {M}_1$ relative to $\mathcal {M}_0$, is defined as
	$$B_{10} = \frac{l({\varvec{y}} \mid \mathcal {M}_1)}{l({\varvec{y}} \mid \mathcal {M}_0)} $$

This desire for greater personal involvement in the process of statistical modeling can be attributed to the seamless integration of subjective beliefs within a Bayesian framework (e.g., McElreath, 2020). However, it is also an overt reaction to past scientific practices in which statistical methods and inference rules were very often deployed without much reflection (see Gigerenzer, 2004, 2018). This reaction against ‘mindless statistics’ is evidenced by the ongoing efforts towards better understanding the different forms of Bayesian inference as well as their standing relative to classical-frequentist inference (for a small selection of recent work, see Douven, 2023; Greenland et al., 2016; Heck et al., 2023; Held & Ort, 2018; Hoijtink et al., 2019; Huisman, 2023; Kruschke, 2021; Schad et al., 2023; Schmalz et al., 2023; Sarafoglou et al., 2024; Tendeiro & Kiers, 2019; Tendeiro et al., 2024).

The present work makes two distinct contributions to this growing body of work. We will begin by proposing two alternative families of priors – “*effect-size*” and “*moment*” priors. These two families of priors, which focus on effects of prespecified sizes, can be conveniently applied in the computation of Bayes factors for standard statistical methods. And on top of the desirable properties found in default Bayes factors (e.g., large-sample consistency), the Bayes factors obtained with the new priors also satisfy a number of additional desiderata, such as yielding the equivalent results when testing the same hypothesis in different guises (e.g., *t* test versus ANOVA). To facilitate the computation of Bayes factors using these new families of priors, this manuscript is accompanied by an easy-to-use R package implementing them for a variety of statistical tests.

After this initial proposal, we will take a step back and offer some perspective on how hypothesis testing is pursued by means of Bayes factors vis-à-vis frequentist significance tests. This contrast will lead us to a novel insight, namely that both default Bayes factors and the new Bayes factors proposed here are equivalent to *test-statistic-based Bayes factors* (Johnson, 2005). This equivalence is a quite felicitous one, as test-statistic-based Bayes factors provide a general approach for easily computing Bayes factors for most hypothesis-testing cases in which a frequentist Neyman-Pearson solution is available (i.e., whenever statistical power can be computed as a function of effect size).^1^

## Default priors and their alternatives

Let us begin by considering what is arguably one of the simplest cases to which default priors can be applied – the one-sample *t* test. Here, the null hypothesis $H_0$ is a *simple* hypothesis stating that the population mean $\mu$ of the data is zero, whereas the alternative $H_1$ is a *composite* hypothesis stating that it differs from zero. The assumptions (default priors included) made for models $\mathcal {M}_0$ and $\mathcal {M}_1$ representing $H_0$ and $H_1$, respectively, are summarized in Table 2: The first row describes the assumed data distribution, which is shared by both Bayesian and frequentist approaches to hypothesis testing. The remaining rows refer to the distributional assumptions made for the model parameters. These assumptions are required by the Bayesian approach. The variance parameter $\sigma ^2$, which is found in both models $\mathcal {M}_0$ and $\mathcal {M}_1$ is assigned a vague prior known as Jeffreys ’s (1946) transformation-invariant prior. Model $\mathcal {M}_1$ also includes the standardized effect-size parameter $d = \nicefrac {\mu }{\sigma }$, which is attributed a scaled Cauchy prior – this is Rouder et al.’s (2009) default prior.

From the onset, Rouder and Morey (2012) are explicit about the fact that “these priors are *subjective* [emphasis added] and convey specified prior beliefs about the alternative under consideration” (Rouder & Morey, 2012, p. 883; see also Rouder et al., 2016). This is a crucial point given that Bayes factors are strongly dependent on the choice of priors, especially the prior for the targeted effect (see Du et al., 2019; Rouder & Morey, 2012; Rouder et al., 2009, 2012). This dependency is such that radically different Bayes factors can be obtained for the same data by simply varying the prior chosen (e.g., Du et al., 2019). Given that this subjective choice is highly consequential, researchers are well advised to be judicious.

**Table 2:** Distributional assumptions in a default Bayesian one-sample *t* test

Variable	$\mathcal {M}_0$	$\mathcal {M}_1$
Data $y_i$	$\mathcal {N}(0,\sigma ^2)$	$\mathcal {N}(\sigma d,\sigma ^2)$
Variance $\sigma ^2$	$f(\sigma ^2) \propto \frac{1}{\sigma ^2}$	$f(\sigma ^2) \propto \frac{1}{\sigma ^2}$
Standardized	n.a.	Cauchy(*r*)
Effect size $d =\frac{\mu }{\sigma }$		

*Note* $$\mathcal {N}=$$normal distribution; n.a. = not applicable; Cauchy$$(r) = $$ scaled Cauchy distribution with scale factor *r*. The mean $$\mu $$ is expressed as $$\sigma d$$ in $$\mathcal M_1$$, using the standardized effect-size *d* and the standard deviation $$\sigma $$, for the sake of consistency with subsequent tables

**Fig. 1:**
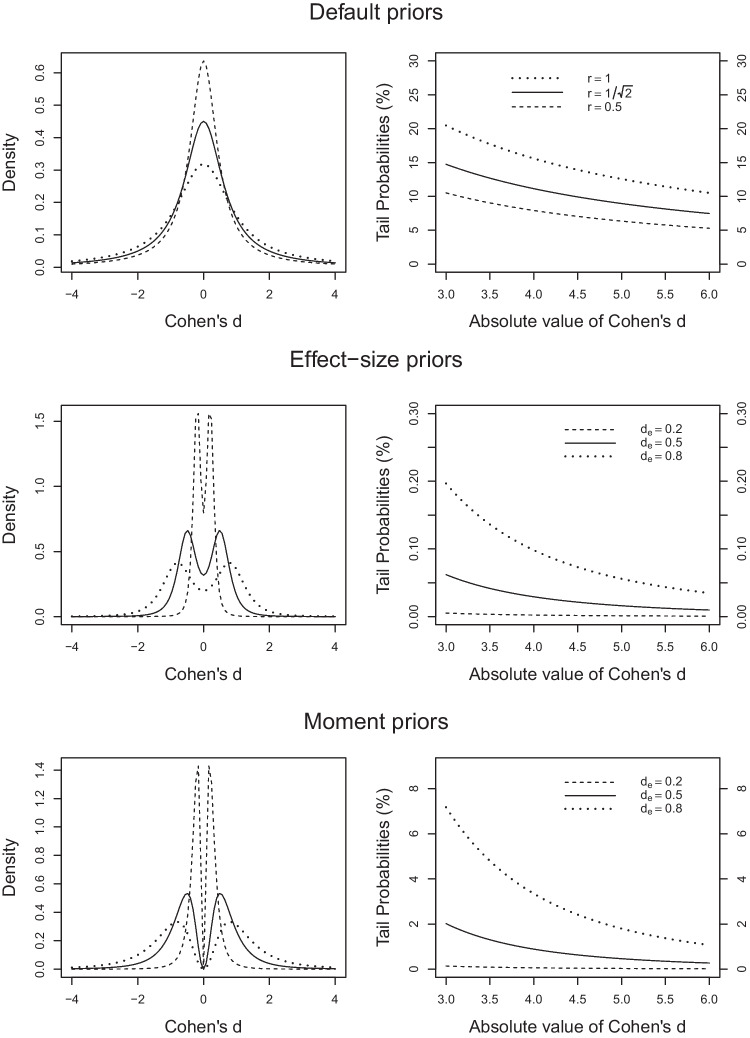
Densities and tail probabilities under default, effect-size, and moment priors. *Note.* Densities (*left panels*) and tail probabilities (*right panels*) of effect sizes under default (Cauchy) priors (*upper panels*) for small-, medium-, and large-scale factor *r* as shown on the *top-right panels*, effect-size priors (*middle panels*) for small, medium, and large effects $d_e$ in terms of Cohen’s *d*, and moment-priors (*lower panels*) for these effect sizes. Parameters for effect-size priors and moment priors and scale factor are set to the values recommended in the section “How to choose degrees of freedom and scale factor?”

Aside from facilitating the computation of Bayes factors, the default priors also satisfy a number of desirable theoretical consistency conditions, which will be elaborated below (Rouder & Morey, 2012; Rouder et al., 2009, 2012; Wetzels et al., 2012). That being said, they also possess features that strike us as undesirable:

1. One of the goals of the default prior on effect sizes is to capture the beliefs surrounding the alternative hypothesis $H_1:$ $\mu \ne 0$. However, this goal is arguably undermined by the fact that the value $\mu = 0$ is always established as *the single most likely value* (Pramanik & Johnson, 2024). This feature is illustrated in the upper left panel of Fig. 1.
2. The default prior includes a scaling parameter *r* that can be adjusted as a function of one’s beliefs regarding effect-size magnitude. The upper left panel of Fig. 1 illustrates the default priors for the *r* values commonly chosen for small, medium, and large effect sizes. The upper right panel shows how likely large effects are assumed to be under the default priors. As can be seen, the default priors tend to assign considerable probability mass to unrealistically large effect sizes (cf. Rouder et al., 2009, p. 230). For example, in the case of a small scaling factor $r = 0.5$, the probability mass for absolute effects larger than 6 is still above 5%.
3. The two previous issues compound when attempting to set up a default prior that reflects the expectation of a moderate or a large effect, as a considerable amount of mass is assigned to both small and extremely large effects. For example, for the case of scale factor $r = 1$, illustrated in the upper left panel of Fig. 1, the probability mass assigned to absolute effects *d* between 0 and 0.4 is 24%. In turn, the probability mass assigned to absolute effects larger than 6 is 10.5%.

At this point, it is appropriate to acknowledge that there are situations in which Feature 1’s presumption that, the smaller an effect in absolute size, the more likely it is, makes much sense. For example, if there truly is an effect of extrasensory perception, it is likely very small; otherwise, it would already have been established beyond doubt. Similarly, if well-established laws of physics are empirically inadequate, then the size of the discrepancies must be extremely small; otherwise, they would have been detected by now. In such cases, a prior according to which effects are the more likely the smaller they are may be justified although one would want it to have much less heavy tails than the default priors (Feature 2; see also van Ravenzwaaij & Wagenmakers, 2022, pp. 459–460, for a related discussion, and Berger et al., 1997, for a discussion of these cases in relation to the null hypothesis). Furthermore, the default priors can also be said to be centered on a given effect size in terms of the median rather than the mode since the median of the absolute effect size under a scaled Cauchy prior with scale factor *r* equals *r* (Mulder, 2023): Fifty percent of effect sizes are a priori assumed to be smaller, and 50% larger in absolute value.^2^

But what is the actual impact of these features on the Bayes factors that are ultimately computed? As a first example, consider the impact of using a prior that places substantial probability mass over extreme effect sizes. Data with smaller, realistic true effect sizes are extremely unlikely if assumed to arise from a distribution with a much larger underlying effect. This will reduce the model’s marginal likelihood, which can be thought of as the likelihood of the data averaged over a range of effect sizes weighted by their respective prior. This reduction is due to the prior placing considerable weights on more extreme effects. All of this implies that the marginal likelihood for $\mathcal {M}_1$, $l({\varvec{y}} \mid \mathcal {M}_1)$, will be smaller under the default prior than under some alternative prior distribution that places less probability mass on extreme effect sizes. The end result is a Bayes factor $B_{10} = \nicefrac {l({\varvec{y}} \mid \mathcal {M}_1)}{l({\varvec{y}} \mid \mathcal {M}_0)}$ that is more favorable toward the null hypothesis $H_0$ (Rouder et al., 2009; see also Bayarri et al., 2012, Criterion 1, for a related discussion).

Next, consider the impact of the prior density values close to the null effect value $d = 0$. When dealing with real-world sample sizes, the problem of discriminating between $\mathcal M_0$ and $\mathcal M_1$ through data becomes more difficult the more similar their priors are.^3^ That is, it becomes a considerable challenge for the Bayes factor to yield decisive evidence for or against $H_0$ whenever the prior heavily weighs values close to zero.

As noted by Tendeiro and Kiers (2019), “all available software packages that allow computing Bayes factors do offer default within-model priors. In this sense, practitioners have little choice than to use one of the default options” (p. 781).^4^

Ideally, researchers should have available in their toolboxes alternative families of priors that mitigate the kinds of issues discussed above while retaining all of the desirable features that made default priors so popular to begin with. The first goal of this paper is to deliver that. We propose two families of priors, which were developed with the following goals in mind:

1. Use vague, “objective” priors for parameters outside the hypotheses tested (e.g., the $\sigma ^2$ parameter in the *t* test).
2. Focus the prior distribution of effects on effects of a prespecified absolute effect size so that effects of this size are the most likely ones under the prior.
3. Use distributions with less heavy tails than the Cauchy distribution.
4. Use a uniform prior distribution on the set of effect parameters of the prespecified effect size.^5^

As before, let us consider the simple case offered by the one-sample *t* test. For a given *prespecified effect size* $d_e$, there are two effects that represent that effect size, namely the effect $d_e$ and the effect $-d_e$. As per goal 4 above, these receive equal weight.

The first family of alternative priors, which we term *effect-size priors*, uses equal-weight mixtures of two distributions of standardized effect sizes, one centered on $d_e$, the other one centered on $-d_e$. To alleviate the concern over excessively heavy tails, the mixture components are scaled *t* distributions with degrees of freedom $\nu$ larger than one (with $\nu =1$ corresponding to the Cauchy distribution). The middle left panel of Fig. 1 illustrates these priors for three prespecified effect sizes $d = d_e$, corresponding to effects traditionally considered small, medium, and large (Cohen, 1988). These prior densities are bimodal and place most probability mass around $-d_e$ and $d_e$. As shown in the middle right panel of Fig. 1, extreme *d* values are now very unlikely (note that one unit on the *y* axis in the middle right panel is only one-hundred of one unit in the upper right panel). This family of effect-size priors is inspired by priors used in the informed Bayesian *t* tests proposed by Gronau et al. (2020).

The second family of priors, which we call *moment priors*, also starts out from *t* distributions for standardized effect sizes *d*. In this case, however, their densities are multiplied by $d^2$ (hence the name “moment priors”). This operation also results in bimodal densities symmetrical around zero. And by scaling these distributions so that the two modes to the left and right of zero coincide with $d_e$ and $-d_e$, these effect sizes are the most likely ones. Under these moment priors, effect sizes much smaller than the prespecified ones are much less likely than under the effect-size priors, with null effects even receiving zero likelihood (see lower left panel of Fig. 1). And as shown in the lower right panel of Fig. 1, extreme *d* values are once again deemed very unlikely (although less so in comparison to effect-size priors). This family of moment priors is inspired by, and in fact a subset of, the nonlocal alternative priors proposed by Johnson and colleagues (Johnson & Rossell, 2010; Pramanik & Johnson, 2024).

Relative to default priors, one way to think of these two families of priors is as ways to improve the sensitivity and specificity for detecting effects of prespecified size (see also Pramanik & Johnson, 2024). Below, we will use these families to develop Bayes factors for one-sample and two-sample *t* tests as well as for regression problems and ANOVA tests. We will discuss key properties of these Bayes factors and apply them to real data. To facilitate their use by researchers at large, we also provide an R package that enables the computation of effect size and moment Bayes factors for all of the statistical tests considered here.^6^

**Table 3:** Distributional assumptions for a Bayesian one-sample *t* test under $\mathcal M_0$, under $\mathcal M_1(E)$ using effect-size priors, and under $\mathcal M_1(M)$ using moment priors

	$\mathcal {M}_0$	$\mathcal {M}_1(E)$	$\mathcal {M}_1(M)$
Data $y_i$	$\mathcal {N}(0,\sigma ^2)$	$\mathcal {N}(\sigma d,\sigma ^2)$	$\mathcal {N}(\sigma d,\sigma ^2)$
Variance $\sigma ^2$	$f(\sigma ^2) \propto \frac{1}{\sigma ^2}$	$f(\sigma ^2) \propto \frac{1}{\sigma ^2}$	$f(\sigma ^2) \propto \frac{1}{\sigma ^2}$
Standardized	n.a.	$f_t(d \mid \nu , \xi , r)$	$\frac{2(\nu -2)}{\nu -1}\frac{d^2}{d_e^2}\times$
effects *d*			$f_t(d \mid \nu , 0, \sqrt{\frac{(\nu -1)d_e}{2\nu } }\>)$
Effects $\xi$ focused on	n.a.	Uniform on the set of $\xi$	n.a.
in effect-size prior		with $|\xi | = d_e$ (i.e., on $\{-d_e, d_e\}$)	

*Note* $$E=$$ effect-size prior; $$M =$$ moment prior; $$\mathcal {N}=$$ normal distribution; n.a. = not applicable; $$f_t(\cdot \mid a, b, c) = $$ density of the scaled and shifted *t* distribution with *a* degrees of freedom, mean *b*, and scale factor *c*; $$d_e = $$ prespecified effect size

The process of coming up with a prior can be guided by formal as well as substantive considerations. When priors are defined solely in terms of desirable formal consistency and invariance conditions, they are sometimes referred to as “objective” priors. One example is Jeffreys ’s (1946) transformation-invariant priors, which often assume counterintuitive shapes. For instance, Jeffreys’s prior for Bernoulli sampling is U-shaped. By contrast, other priors are defined largely in terms of substantive considerations, such as previous data, subjective beliefs, substantive theoretical assumptions, and the like (e.g., Spektor & Kellen, 2018). Such priors are often more narrowly focused on one specific effect pattern.

The priors proposed here combine both types of considerations. In terms of formal constraints, they satisfy desirable consistency and invariance conditions (see Section “Properties of Bayes factors based on effect-size and moment priors”). On the substantive side, they incorporate a preference for effects of a prespecified effect size and a penalty for overly large effect sizes. That said, they do not prioritize any specific effect pattern (e.g., the specific shape of an interaction or, in the case of two-sided *t* tests, the specific direction of an effect) over any other pattern. Without getting into a discussion of which kinds of priors are best (see, e.g., Berger, 2006; Torsen, 2015), we believe that the priors proposed here strike a balance that is quite appealing to any researcher looking for a general solution that does not commit them to strong substantive assumptions.

**Table 4:** Bayes factors for *t* tests and multiple linear regression/ANOVA model comparisons

Prior	Bayes factor $B_{10}$
One-sample *t* test	
Effect Size	$\int _{-\infty }^{\infty } \frac{f_{nct}(t\mid N-1, \sqrt{N}d)}{f_{nct}(t\mid N-1, 0)}\frac{1}{2}\left( f_t(d \mid \nu , -d_e, r) + f_t(d \mid \nu , d_e, r)\right) \text {d}d,$
Moment	$\int _{-\infty }^{\infty } \frac{f_{nct}(t\mid N-1, \sqrt{N}d)}{f_{nct}(t\mid N-1, 0)} \frac{2(\nu -2)}{\nu -1}\frac{d^2}{d_e^2} f_t\left( d\mid \nu , 0, \sqrt{\frac{(\nu -1)d_{e}^{2}}{2\nu }}\right) \text {d}d$
Two-sample *t* test ($N = N_1 + N_2, M = \frac{N_1 N_2}{N})$	
Effect Size	$\int _{-\infty }^{\infty } \frac{f_{nct}(t\mid N-2, \sqrt{M}d)}{f_{nct}(t\mid N-2, 0)}\frac{1}{2}\left( f_t(d \mid \nu , -d_e, r) + f_t(d \mid \nu , d_e, r)\right) \text {d}d$
Moment	$\int _{-\infty }^{\infty } \frac{f_{nct}(t\mid N-2, \sqrt{M}d)}{f_{nct}(t\mid N-2, 0)} \frac{2(\nu -2)}{\nu -1}\frac{d^2}{d_e^2} f_t\left( d\mid \nu , 0, \sqrt{\frac{(\nu -1)d_{e}^{2}}{2\nu }}\right) \text {d}d$
Regression/ANOVA ($$M = N-p)$$	
Effect Size	$\int _{0}^{\infty } \frac{f_F(F \mid q, M-q, M \lambda ^2)}{f_F(F \mid q, M-q, 0)} \frac{\Gamma ((\nu + q)/2)}{\Gamma (\nu /2)\Gamma (q/2)}(\nu r^2)^{\frac{\nu }{2}} (\lambda ^2)^{\frac{q}{2}-1} (\lambda ^2 + f^2 + \nu r^2)^{-\frac{\nu + q}{2}}$
	$$_{2}F_{1}\left( \frac{\nu + q}{4};\frac{1}{4}(2 + \nu + q);\frac{q}{2};\frac{4f^2\lambda ^2}{(\lambda ^2 + f^2 + \nu r^2)^2}\right) \text {d}\lambda ^2 $$
Moment	$\int _{0}^{\infty }\frac{f_F(F \mid q, M-q, M \lambda ^2)}{f_F(F \mid q, M-q, 0)} \frac{2(\nu -2)}{q(\nu +q-2)f^2} \frac{\Gamma ((\nu + q)/2)}{\Gamma (\nu /2)\Gamma (q/2)}$
	$$\left( \frac{2}{(q+\nu -2)f^2} \lambda ^2 \right) ^{\frac{q}{2}} \left( 1 + \frac{2}{(q+\nu -2)f^2} \lambda ^2 \right) ^{-\frac{\nu +q}{2}} \text {d}\lambda ^2$$

*Note* $f_t(\cdot \mid a, b, c) =$ density of the scaled and shifted *t* distribution with *a* degrees of freedom, mean *b*, and scale factor *c*; $f_{nct}(\cdot \mid \nu , \rho ) =$ density of the noncentral *t* distribution with $\nu$ degrees of freedom and noncentrality parameter $\rho$; $f_F(\cdot \mid \nu _1, \nu _2, \rho ) =$ density of the noncentral *F* distribution with $\nu _1$ and $\nu _2$ degrees of freedom and noncentrality parameter $\rho$; $_{2}F_{1}(\cdot ;\cdot ;\cdot ;\cdot ) =$ hypergeometric function (Abramowitz & Stegun, 1972, Chap. 15). All of the Bayes factor formulae are new. Gronau et al. (2020) derived formulae related to the effect-size Bayes factors for *t* tests, and Johnson and Rossell (2010, Eq. 22) derived an alternative formula for the moment Bayes factor

## A bestiary of Bayes factors for effect size and moment priors

### One-sample *t* tests

Table 3 summarizes the assumptions required for a Bayesian one-sample *t* test using the effect size and moment priors. As can be seen in Table 4, the effect size and moment Bayes factors for a one-sample *t* test with prespecified effect size $d_e$ depend on the data only via the test statistic *t*. And just like for default Bayes factors, their computation boils down to a unidimensional integration problem. Indeed, for $\nu = 1$ and $d_e=0$, the effect-size Bayes factor corresponds to Rouder et al. ’s (2009) default Bayes factor. The moment Bayes factor, on the other hand, converges to Pramanik and Johnson ’s (2024) Bayes factor based on non-local alternative priors as $\nu \rightarrow \infty$.

### Two-sample *t* tests

Table 5 lists the assumptions required for a Bayesian two-sample *t* test comparing data $x_i$, $i=1,\ldots ,N_1$ and $y_i$, $i=1,\ldots ,N_2$, using the effect-size and moment priors. Just as in the one-sample case, the effect-size and moment Bayes factors correspond to a one-dimensional integral that solely depends on the *t* statistic (see Table 4). And once again, Rouder et al. (2009) default Bayes factor and Pramanik and Johnson ’s (2024) Bayes factor based on non-local alternative priors correspond to special cases.

**Table 5:** Distributional assumptions for a Bayesian two-sample *t* test under $\mathcal M_0$, under $\mathcal M_1(E)$ using effect-size priors, and under $\mathcal M_1(M)$ using moment priors

	$\mathcal {M}_0$	$\mathcal {M}_1(E)$	$\mathcal {M}_1(M)$
Data $x_i$	$\mathcal {N}(\mu ,\sigma ^2)$	$\mathcal {N}(\mu - \frac{1}{2}\sigma d ,\sigma ^2)$	$\mathcal {N}(\mu - \frac{1}{2}\sigma d ,\sigma ^2)$
Data $y_i$	$\mathcal {N}( \mu ,\sigma ^2)$	$\mathcal {N}(\mu + \frac{1}{2}\sigma d ,\sigma ^2)$	$\mathcal {N}(\mu + \frac{1}{2}\sigma d ,\sigma ^2)$
Mean $\mu$	$f(\mu ) \propto 1$	$f(\mu ) \propto 1$	$f(\mu ) \propto 1$
Variance $\sigma ^2$	$f(\sigma ^2) \propto \frac{1}{\sigma ^2}$	$f(\sigma ^2) \propto \frac{1}{\sigma ^2}$	$f(\sigma ^2) \propto \frac{1}{\sigma ^2}$
Standardized		$f_t(d \mid \nu , \xi , r)$	$\frac{2(\nu -2)}{\nu -1}\frac{d^2}{d_e^2}\times$
effects *d*	n.a.		$f_t( d \mid \nu , 0,\sqrt{\frac{(\nu -1)d_e}{2\nu } }\>)$
Effects $\xi$ focused on	n.a.	Uniform on the set of $\xi$	n.a.
in effect-size prior		with $|\xi | = d_e$ (i.e., on $\{-d_e, d_e\}$)	

*Note* $E=$ effect-size prior; $M =$ moment prior; $\mathcal {N}=$normal distribution; n.a. = not applicable; $f_t(\cdot \mid a, b, c) =$ density of the scaled and shifted *t* distribution with *a* degrees of freedom, mean *b*, and scale factor *c*; $d_e =$ prespecified effect size

### Multiple linear regression

In a multiple regression model, *N* observations $${\varvec{y}} = (y_1,\ldots ,y_N)^t$$ are predicted from *k* predictors $${\varvec{x}}_1 =(x_{11},\ldots ,$$ $$x_{N1})^t,$$ $${\varvec{x}}_2 = (x_{12},\ldots ,x_{N2})^t,$$ $$\ldots , {\varvec{x}}_k = (x_{1k},\ldots ,x_{Nk})^t$$ by means of regression weights $$\beta _j$$ via a linear equation:

2 $$\begin{aligned} \hat{y}_i = \beta _1 x_{i1} + \beta _2 x_{i2} + \ldots + \beta _k x_{ik}, \quad i = 1, \ldots , N, \end{aligned}$$

where $\hat{y}_i$ are the predicted values, and the residuals, $y_i - \hat{y}_i$, are assumed to follow a normal distribution with mean 0 and variance $\sigma ^2$. Frequently, the first predictor ${\varvec{x}}_1$ has values of one for each observation, ${\varvec{x}}_1 = (1,\ldots ,1)^t$, in which case $\beta _1$ is called the intercept.

Collecting the predictor variables as columns in a matrix $X = ({\varvec{x}}_1,\ldots {\varvec{x}}_k$) and regression weights in a vector ${\varvec{\beta }} = (\beta _1,\ldots ,\beta _k)^t$, Equation 2 can be written compactly as

3 $$\begin{aligned} {\varvec{\hat{y}}} = X {\varvec{\beta }}. \end{aligned}$$

Hypothesis tests in the context of regression models contrast a reduced model, in which one or more of the regression weights are set zero, with the full model, in which all regression weights are allowed to differ from zero. Let the first *p* regression weights be those that are retained for the reduced model collected in ${\varvec{\beta }}_1 = (\beta _1,\ldots ,\beta _p)^t$ and the remaining $q = k-p$ regression weights those that are set to zero in the reduced model ${\varvec{\beta }}_2 = (\beta _{p+1},\ldots ,\beta _{k})^t$. The null hypothesis can then be stated as $H_0 : {\varvec{\beta }}_2 = \varvec{0}$, the alternative as $H_1 : {\varvec{\beta }}_2 \ne \varvec{0}$, where $\varvec{0}$ is a vector with all component values equal to zero.

#### Effect size in multiple regression

Let the predictors associated with regression weights ${\varvec{\beta }}_1$ be collected as the columns of matrix $X_1$ and those associated with ${\varvec{\beta }}_2$ as the columns of matrix $X_2$, such that $X = (X_1,X_2)$. The effect-size measure associated with the above hypothesis test is Cohen’s $f^2$, which can be understood as the standardized noncentrality parameter of the *F* distribution in comparing full and reduced models. This hypothesis test is equivalent to one in which we first partial out the predictors $X_1$ from each of the predictors collected in $X_2$ and then test for the role of the predictors $X_2$ (after partialing out $X_1$) in predicting the residuals of the data under the reduced model. Of the *N* residuals, *p* are redundant so that the effective sample size in this contrast is $N-p$. And therefore, we standardize the effect size $f^2$ as the noncentrality parameter divided by the effective sample size.^7^

The noncentrality parameter is the squared Euclidean norm of the vector of standardized regression weights ${\varvec{\beta }}^*_2$, after standardization by the variance-covariance matrix of ${\varvec{\beta }}_2$ that applies when the predictors $X_1$ have been partialed out from data and predictors $X_2$ (see Seber, 2015, Chap. 4). This variance-covariance matrix is $\sigma ^2 \Sigma ^{-1}$, with $\Sigma = X_2^t(I - X_1(X_1^t X_1)^{-1} X_1^t)X_2$, where *I* is the identity matrix. Setting ${\varvec{\mu }} = \frac{1}{\sigma } {\varvec{\beta }}_2$, and $\Omega = \Sigma /(N-p)$ it follows that

4 $$\begin{aligned} f^2 = {\varvec{\mu }}^t \Omega {\varvec{\mu }}. \end{aligned}$$

#### Distributional assumptions and Bayes factor

Using the above notation, we can now summarize the distributional assumptions for the Bayesian version of the hypothesis test in Table 6. Note that the effects $\tilde{\varvec{\beta }}_2$ in that table have already been standardized by $\sigma ^{-1}$, but not by $\Sigma$. Just like for the *t* tests considered above, the distribution of effects is still based on a *t* distribution with $\nu$ degrees of freedom and a scale factor *r* – but now it is a multivariate *t* distribution (Kotz & Nadarajah, 2004). As explained by Wetzels et al. (2012), the factor $N-p$ in the scale matrix of this distribution implies that “the prior is 1/nth [1/(N-p)th; see Footnote 7] as important as the sample” (p. 106). For this reason, such priors are often called unit information priors because the prior exerts, roughly speaking, as much influence as one data point (remember that the effective sample size is $N-p$).

**Table 6:** Distributional assumptions for a Bayesian test under $\mathcal M_0$ (reduced model), under $\mathcal M_1(E)$ using effect-size priors and under $\mathcal M_1(M)$ using moment priors

	$\mathcal {M}_0$	$\mathcal {M}_1(E)$	$\mathcal M_1(M)$
Data $\varvec{y}$	$\mathcal {N}(X_1{\varvec{\beta }}_1,\sigma ^2 I)$	$\mathcal {N}(X_1{\varvec{\beta }}_1 + \sigma X_2\tilde{\varvec{\beta }}_2,\sigma ^2 I)$	$\mathcal {N}(X_1{\varvec{\beta }}_1 + \sigma X_2\tilde{\varvec{\beta }}_2,\sigma ^2 I)$
Regression weights ${\varvec{\beta }}_1$	$f({\varvec{\beta }}_1) \propto 1$	$f({\varvec{\beta }}_1) \propto 1$	$f({\varvec{\beta }}_1) \propto 1$
Variance $\sigma ^2$	$f(\sigma ^2) \propto \frac{1}{\sigma ^2}$	$f(\sigma ^2) \propto \frac{1}{\sigma ^2}$	$f(\sigma ^2) \propto \frac{1}{\sigma ^2}$
Effects $\tilde{\varvec{\beta }}_2 = \nicefrac {{\varvec{\beta }}_2}{\sigma }$	n.a.	$f_t(\tilde{\varvec{\beta }}_2 \mid \nu ,\varvec{\mu }, r^2\Omega ^{-1})$	$\frac{2(\nu -2)}{q(\nu + q -2)f^2}\tilde{\varvec{\beta }}_2^t \Omega \tilde{\varvec{\beta }_2} \times$
			$$f_t(\tilde{\varvec{\beta }}_2 \mid \nu , \varvec{0}, \frac{(\nu + q -2)f^2}{2\nu }\Omega ^{-1})$$
Effects ${\varvec{\mu }}$ focused on	n.a.	Uniform on the set of	n.a.
in effect-size prior		${\varvec{\mu }}$ with $\sqrt{ {\varvec{\mu }}^t \Omega {\varvec{\mu }}} = f$	

*Note* $\mathcal {N}=$normal distribution; n.a. = not applicable; $f_t(\cdot \mid a, \varvec{b}, C)=$ density of scaled and shifted multivariate ($q-$dimensional) *t* distribution with *a* degrees of freedom, mean vector ${\varvec{b}}$ and scale matrix *C*

Table 4 shows the formulae for the effect-size and moment Bayes factors. Once again, these Bayes factors can be computed as one-dimensional integrals, and they depend on the data solely via the value of the test statistic, in this case *F*. For the special case of $\nu = 1$, $f^2=0$, and $p=1$, the effect-size Bayes factor corresponds to Rouder et al.’s (2009) default Bayes factor, which contrasts an intercept-only model with a model that includes *q* predictors, with the exception that we divide $\Sigma$ by $N-p = N-1$ instead of *N*.

### ANOVA

ANOVA models and regression models are both members of the family of general linear models. The Bayes factors for tests of main effect and/or interactions using effect-size or moment priors are therefore the same as those used for comparing regression models (see Table 4). However, the effect size for ANOVA tests is conventionally expressed in terms of *f* instead of $f^2$. In the Supplementary Materials, we briefly sketch how ANOVA analyses with between-participants and within-participant factors can be framed as regression analyses (e.g., Judd et al., 2017; Maxwell & Delaney, 2004; but see Gelman, 2005, for complicated ANOVA designs in which it is far from trivial to find the right way to do this).

As before, computing effect-size and moment Bayes factors boils down to a one-dimensional integration problem. This contrasts with the default Bayes factor, which requires higher-dimensional integration (see Rouder et al., 2012).^8^ That being said, Rouder and Morey (2012) framework is somewhat more flexible in that random factors (for factors over and above the person factor) can also be dealt with.

## Properties of Bayes factors based on effect-size and moment priors

The literature on hypothesis testing and model selection with Bayes factors highlights a number of properties that they should possess (e.g., Jeffreys, 1942; Rouder et al., 2012; see also Bayarri et al., 2012). We discuss each of these properties in turn.

### Scale invariance

The value of the Bayes factor should not change if the observations are multiplied by a constant (other than zero). Effect-size and moment Bayes factors satisfy this demand because the effect size (*d*, *f*, or $f^2$) is scale-invariant, as is the value of the statistic (*t* or *F*) through which the Bayes factor depends on the data.

### Large-sample consistency

If the true effect is zero, then the Bayes factor $B_{10}$ should converge to zero as sample size *N* increases. In contrast, it should go to infinity when the true effect differs from zero. Bayes factors are known to satisfy consistency under mild regularity conditions (Chib & Kuffner, 2016)^9^, and the effect-size and moment Bayes factors are no exception. That being said, formal proofs of this property usually assume that the priors assign positive likelihood to all values. The fact that the moment priors assign zero likelihood to null effects therefore requires some additional consideration (see Supplementary Materials).

### Consistency in information

Even for fixed *N*, the Bayes factor should show unlimited evidence for the alternative model as the respective test statistic increases. In other words, for fixed *N*, the Bayes factor $B_{10}$ tends to infinity as *t* or *F* go to infinity. As shown in the Supplementary Materials, consistency in information is given for the effect-size Bayes factors for one-sample *t* tests if $N \ge 1 + \nu$, for two-sample *t* tests if $N\ge 2 + \nu$, and for hypothesis tests in regression and ANOVA if $N\ge p + q + \nu$. It is given for the moment Bayes factors for one-sample *t* tests if $N \ge \nu - 1$, for two-sample *t* tests if $N\ge \nu$, and for hypothesis tests in regression and ANOVA, if $N\ge p + q + \nu -2$.^10^

The fact that the number of data points minimally required for consistency in information depends on the $\nu$ degrees of freedom of the prior accounts for the observation that a normal prior on effects does not achieve consistency in information. After all, the normal distribution corresponds to the limit of *t* distributions as $\nu \rightarrow \infty$. And given that Pramanik and Johnson ’s (2024) Bayes factors for *t* tests based on nonlocal alternative priors are obtained from the moment Bayes factors as $\nu \rightarrow \infty$, it follows that they do not satisfy consistency in information.

### Predictive matching

If the data are uninformative regarding the comparison of $H_0$ and $H_1$, then their marginal likelihoods should be the same, which entails a Bayes factor of 1. Because improper priors are attributed to the parameters of the reduced model, Bayes factors can only be computed for data for which the reduced model can be estimated (i.e., for which $X_1$ has rank *p*), implying that we need at least $N\ge p$ data points before we can consider Bayes factors.

Uninformative data are given if, for example, we have only one data point from each group in a two-sample *t* test. This means that the data do not provide the redundancy required to estimate the variability of the residuals under the full model underlying the test. In general, this is the case if there are no more than $p + q$ data points (i.e., $N \le p + q)$ if *X* has full rank.

Another example of uninformative data is one where we have possibly many data points from one group in a two-sample *t* test, but no data from the other group. This means that we can estimate the reduced model, but not the full model. In general, this case arises if we have *N* data points with design matrix $X_1$ being of rank *p*, but with the full design matrix $X = (X_1,X_2)$ also of rank *p* so that the data contain no information regarding the full model that cannot already be accounted for by the reduced model.

In the Supplementary Materials, we show that effect-size and moment Bayes factors satisfy predictive matching in these cases, such that $B_{10}=1$. Predictive matching is in general not satisfied by informed priors such as those considered by Gronau et al. (2020) for *t* tests.

### Framing invariance

We took the liberty to introduce a fifth desirable criterion, *framing invariance*. Framing invariance is a property of a collection of Bayes factors capable of testing the same hypothesis in different guises. For example, it is well known that a dependent-measures *t* test is equivalent to

- a one-sample *t* test on the difference between the measures,
- an ANOVA with within-participant factor “measure” and a test for an effect of that factor,
- an ANOVA on the differences and a test for a significant intercept in that model,
- a regression analysis contrasting a reduced model with *S* predictors effect-coding the *S* participants and a full model with an additional predictor for the factor “measure”, and
- a regression analysis on the difference score with a reduced model with $p=0$ predictors and a full model that contains an intercept.

Similarly, a two-sample *t* test can equivalently be performed as an ANOVA testing for the main effect of a group factor and as a regression analysis with an intercept in the reduced model and a factor coding group membership in the full model.

All of these analyses test the same hypotheses with the same distributional assumptions on the data, and most researchers would probably expect the same Bayes factors to be found irrespective of how the hypothesis test is framed, once differences in scaling and effect size computations are taken into account (e.g., *d* in *t* tests needs to be transformed into *f* values for ANOVA models and $f^2$ values for regression models).

We call a set of Bayes factors with this property “framing invariant”. Note that the default Bayes factors as implemented in the BayesFactor package (Morey and Rouder, 2013) and JASP (Love et al., 2019); Wagenmakers et al., 2018) do not always satisfy framing invariance. For example, the Bayes factor for a repeated-measures ANOVA with a single two-level factor is not equivalent to the Bayes factor for a one-sample *t* test on the personwise differences between the two factor levels. This difference is a consequence of the fact that the default priors used under the different framings are not always compatible with each other.

As shown in the Supplementary Materials, the two families of Bayes factors proposed here satisfy framing invariance. For an effect size *d* in a one-sample *t* test, the appropriate *f* and $f^2$ in equivalent ANOVA and regression analyses are $f = d$ and $f^2 = d^2$. To establish equivalence between a two-sample *t* test and equivalent ANOVA and regression models, two points need to be kept in mind:

i) The standardization of the effect-size measure relies on the effective sample size of $N-p =N-1$ in equivalent ANOVA and regression models and on *N* in the two-sample *t* test.
ii) Effect sizes in ANOVA and regression models are not only standardized in terms of $\sigma ^2$, the population variance in the dependent variable, but also in terms of the variance-covariance matrix $\Sigma$ of the predictors.

Taken together, this means that equivalence is achieved if *d* for the effect-size and moment prior of the two-sample *t* test and *f* and $f^2$ for the effect-size and moment priors in ANOVA and regression models, respectively, stand in the following relationship: $d = \sqrt{(N-1)/M} f$, where $M = N_1 N_2 / (N_1 + N_2)$.^11^

## How to choose degrees of freedom and scale factor?

**Fig. 2:**
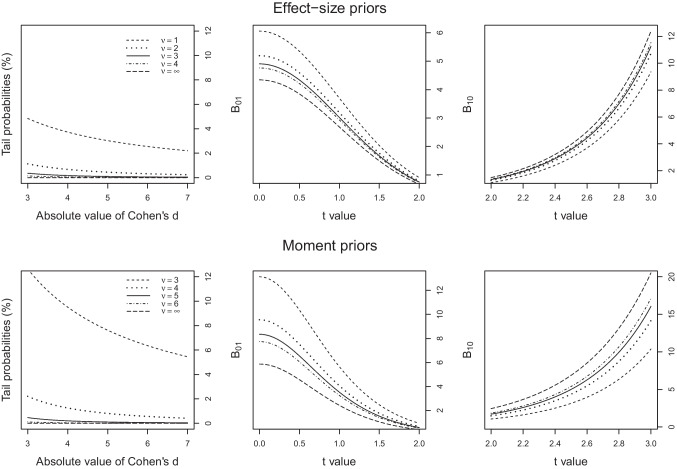
Tail probabilities and Bayes factor values for effect-size and moment priors for different degrees of freedom $\nu$. *Note*. Complementary cumulative probabilities (*left panel*) of effect sizes, values of $B_{01}$ (*middle panel*) for *t* values between 0 and 2 and $B_{10}$ for *t* values between 2 and 3 under effect-size priors with $d_e = 0.3$ and $r = 0.5$ (*upper panels*) and moment priors with $d_e = 0.3$ (*lower panels*) for different degrees of freedom $\nu$

The purpose of increasing the degrees of freedom $\nu$ from $\nu = 1$ implicit in the default Bayes factor is to render large effect sizes less likely. The upper left panel of Fig. 2 shows the likelihood of large effect sizes for effect-size priors with $d_e = 0.3$ and $r=0.5$ for degrees of freedom given by $\nu \in \{1, 2, 3, 4, \infty \}$. Note that the prior with $\nu = \infty$ is a normal prior. Evidently, the issue is quickly mitigated by increasing the degrees of freedom. As exemplified in Fig. 2, a value of $\nu = 3$ appears to realize a reasonable compromise in which the likelihood of extreme values is strongly reduced while rare occurrences of very large effect sizes are still accounted for; going from $\nu = 3$ to $\nu =4$ seems to have little impact.

The upper middle panel of Fig. 2 shows the Bayes factor $B_{01} = 1/B_{10}$ gauging the evidence for $H_0$ relative to $H_1$ for these priors, for a one-sample *t* test on $N = 50$ data points and values of *t* between 0 and 2. As can be seen, increasing the degrees of freedom leads to a decrease in the preference for the null: The size of the successive decrements decreases quickly and again, the differences between $\nu = 3$ and $\nu = 4$ are negligible.

The upper right panel of Fig. 2 shows $B_{10}$ gauging the evidence for $H_1$ relative to $H_0$ for values of *t* between 2 and 3. As can be seen, increasing degrees of freedom increases the evidence for the alternative in steps that are sizeable for $\nu <3$, but marginal for $\nu \ge 3$. These patterns are representative across values of *r* and $d_e$. All things considered, we recommend the use of $\nu = 3$.^12^

**Fig. 3:**
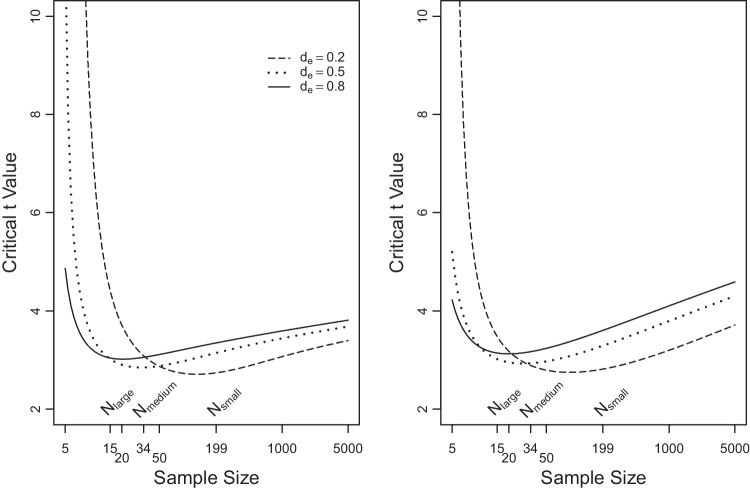
Values of *t* required to achieve a Bayes factor of 10 for effect-size (*left panel*) and moment (*right panel*) Bayes factors. *Note*. The *horizontal axis* is on a logarithmic scale

The lower panels of Fig. 2 show the analogous information for moment priors. Based on the same considerations, our recommendation here is to set $\nu = 5$. However, note that as *q* increases in regression and ANOVA designs, the moment priors imply increased probabilities for large effect sizes. This reflects the fact that the moment priors multiply the multivariate *t* density by a quadratic term, $\varvec{\tilde{\beta }}_2^t \Omega \varvec{\tilde{\beta }}_{2}$, which increases the relative likelihood of large effects $\tilde{\varvec{\beta }}_2$. Moreover, the impact of the quadratic term increases as the dimensionality *q* of the effects $\tilde{\varvec{\beta }}_2$ increases. We view this feature of the moment priors as a conceptual disadvantage relative to the effect-size priors, but it can be counteracted to some extent by increasing the degrees of freedom $\nu$ as a function of *q*. We found that for values of $q\le 20$, the tail probabilities for large effect sizes induced by the moment priors remain low, if $\nu$ is set to $5 + (q-1)$. For rare cases with $q>20$, even larger degrees of freedom should be chosen based on the tail probabilities, which also diminishes the ease with which moment priors can be used.

Turning to the choice of the scale factor *r* in effect-size priors, note that with $\nu \ge 3$ we can consider variances of the effect-size priors. The effect-size priors are mixtures with variances that are increased relative to the variance of each mixture component due to the spread induced by combining two distributions with different modes. The choice of *r* determines the variance of each mixture component, which is $\frac{\nu }{\nu -2}r^2$. The variance due to the spread between the two mixture components amounts to $d_e^2$. The total variance of the mixture distribution is therefore $\frac{\nu }{\nu -2}r^2 + d_e^2$. We recommend choosing *r* so that both contributions to the total variance are equally large. In other words, we recommend setting $r = \sqrt{\frac{\nu -2}{\nu }} d_e$. For $\nu = 3$, the likelihood of effects between $-d_e$ and $d_e$ is thereby 50% and of effects between $-2d_e$ and $2d_e$ 96%. In words, this setting expresses the belief that underlying effects should largely fall in a range from zero to twice the targeted effect size in either direction.

**Table 7:** Default, effect-size, and moment Bayes factors for *t* tests

		Effect-size BF			Moment BF		
*t*(79)	Default BF	small	medium	large	small	medium	large
2.03	0.64	2.52	0.98	0.51	2.13	0.58	0.19
2.24	0.98	3.70	1.58	0.80	3.26	0.99	0.34

*Note* BF = Bayes factor

Choosing *r* in this way also implies that the prior variance tracks the magnitude of the targeted effect $d_e$, such that the former is reduced when the latter is smaller. This behavior seems natural and desirable to us given that we want resolution to increase if we look for small effects: If we target a small effect $d_e = 0.2$, we would be more surprised to find one 0.5 units larger, that is, an effect of $d_e = 0.7$, than if we target a large effect of 0.8 and find one 0.5 units larger, that is, an effect of $d_e = 1.3$. Regarding the multivariate effect-size priors used for regression and ANOVA designs, the analogous considerations lead to the recommendations to set $\nu = 3$ and $r = \sqrt{\frac{\nu -2}{\nu q}} f$.

Another interesting consequence of letting *r* depend upon $d_e$ in the effect-size priors is that, as $d_e$ decreases, the effect-size prior becomes more similar to the prior under $H_0$, which concentrates all probability mass on 0. Similarly, the moment priors focus more probability mass on small effect sizes as $d_e$ decreases. As already discussed above, this makes it more difficult for the Bayes factor to discriminate between $\mathcal M_1$ and $\mathcal M_0$ so that larger samples are required to obtain decisive evidence for or against these models. This behavior can be seen in Fig. 3, which plots the *t* value producing a Bayes factor $B_{10}$ of 10 as a function of sample size for the effect-size priors (left panel) and the moment priors (right panel) with small, medium, and large effect. The prior focusing on small effects initially requires larger *t* values for a Bayes factor of 10 than the priors focusing on larger effects, but as the sample size increases its associated Bayes factor is the most sensitive one in the sense that it now requires smaller *t* values to reach Bayes factors of 10 than the other priors. The figure also shows the sample sizes $N_\text {small},$ $N_\text {medium},$ and $N_\text {large}$ computed by means of a power analysis. They are the sample sizes required to detect, in order, a small, medium, and large effect size with significance level $\alpha = .05$ and test power $1-\beta =.80$. As can be seen, the effect-size and moment priors with small, medium, and large effect sizes are each most sensitive for sample sizes in the vicinity of, in order, $N_\text {small}$, $N_\text {medium}$, and $N_\text {large}$.

Lastly, we wish to emphasize that, given the subjective elements in the priors, it is perfectly legitimate for users to decide to use other $\nu$ and *r* values than those recommended here. Depending on the consensus among researchers in a given field of inquiry, there may be good and even intersubjectively agreed-upon reasons for deciding against adopting these recommendations. With that in mind, the R package offered alongside this manuscript allows the user to override our recommendations and specify other values for $\nu$ and *r*.

## Applications

For the sake of comparison, we revisit some applications reported in papers on default Bayes factors.

### *t* tests

Rouder et al. (2009) considered an experiment by Grider and Malmberg (2008) in which positive words were remembered better than neutral ones ($t(79) = 2.24)$ and negative words better than neutral ones (*t*(79)= 2.03). Under the default Bayes factor with $r=1$, the Bayes factors associated with these *t* values, $B_{10} = 0.98$ and $B_{10} = 0.64$, respectively, actually favor the null hypothesis. After computing default Bayes factors with a very small value of *r*, $r=0.1$, (as well as with a standard normal distribution as prior), Rouder et al. (2009) concluded that: “For Grider and Malmberg’s data, any reasonable prior leads to the same conclusion, that the evidence does not support a preference [for $\mathcal M_1$].” (p. 233). As can be seen in Table 7, effect-size and moment Bayes factors focusing on large effects similarly favor $\mathcal M_0$. If small effects are expected a priori, the latter Bayes factors do, however, justify a preference for $\mathcal M_1$, albeit not a very strong one.

**Fig. 4:**
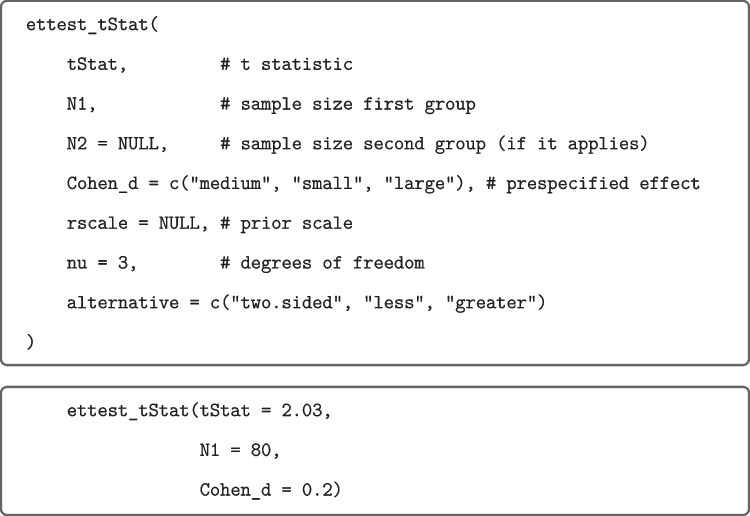
R Function ettest_tStat (*upper panel*) and application (*lower panel*) to data from Grider and Malmberg (2008). *Note.* If rscale and/or nu are not specified, then they are set internally to the recommended values. Moment Bayes factors can be obtained from the analogous R function mttest_tStat included in the R package

The values based on effect-size priors were computed by means of calls to the R function ettest_tStat from our R package as shown in Fig. 4 for the above $t(79)=2.03$ focused on small effects.

### Regression models

Rouder and Morey (2012) applied the default Bayes factor for regression models to a data set by Bailey and Geary (2009) in which the cranial capacity of 175 hominid skulls was regressed on four predictors: (a) local climate variation, (b) global average temperature, (c) parasite load, and (d) the population density.

The models considered by Rouder and Morey (2012) included a full model $\mathcal M_\text {f}$ with these predictors and an intercept, a null model $\mathcal M_0$ including only the intercept, and all 14 reduced models, labeled $\mathcal M_1$ to $\mathcal M_{14}$, including an intercept and a (proper non-empty) subset of the four predictors. The default Bayes factor revealed decisive evidence against the null model for all non-null models, and the current effect-size and moment Bayes factors concur.

Rouder and Morey (2012) also present Bayes factors for the non-null models relative to the full model. These showed decisive evidence in favor of the full model for all but three reduced models, and the effect-size and moment Bayes factors again concur. The results for the remaining three reduced models, $\mathcal M_2$, $\mathcal M_4$, and $\mathcal M_9$, are shown in Table 8. For each of these, the table presents partial $R^2$ for the full model relative to the reduced model, the default Bayes factor, effect-size, and moment Bayes factors for small, medium, and large effects according to Cohen (1988, Chap. 9). These are the Bayes factors $B_{01} = \nicefrac {1}{B_{10}}$ for the preference for the reduced model relative to the full model.

The effect-size priors assuming large effects yield similar conclusions as the default Bayes factors, but the preference for the three reduced models shown by default priors is sometimes much less pronounced for effect-size priors focusing on effects of small or medium size. Considering the moment Bayes factors, the preference shown for the three reduced models is smaller than for default Bayes factors in the case of moment Bayes factors focusing on small effects, but much larger in the case of moment Bayes factors focusing on large effects.

**Table 8:** Default, effect-size, and moment Bayes factors for reduced models against the full models

				Effect-size BF			Moment BF		
	Model	$R^2_p$	Default BF	small	medium	large	small	medium	large
$\mathcal M_2$	L+G+D	.0126	4.41	1.00	3.48	5.92	1.36	8.49	25.30
$\mathcal M_4$	G+P+D	.0002	12.97	3.36	11.76	18.71	6.37	66.98	218.63
$\mathcal M_9$	G+D	.0136	61.03	1.66	14.77	41.07	3.26	49.26	222.66

**Fig. 5:**
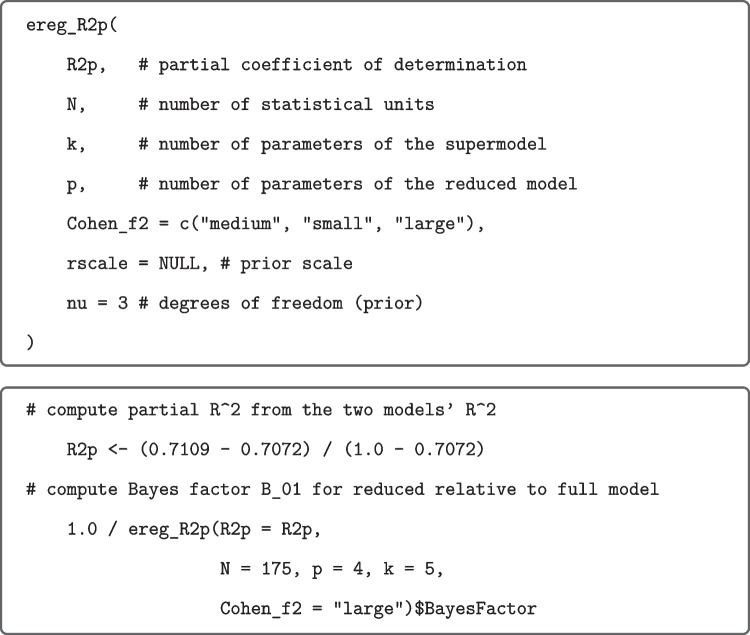
R Function ereg_R2p (*upper panel*) and application (*lower panel*) to data from Bailey and Geary 2009. *Note.* If rscale and/or nu are not specified, they are set internally to the recommended values. The models have one more parameter than they have predictors due to the inclusion of an intercept in each model. Moment Bayes factors can be computed using the analogous function mreg_R2p in our R package. There are also functions ereg_FStat and mreg_FStat proceeding from *F* values, as well as the functions ereg_tStat and mreg_tStat proceeding from *t* values when testing a single predictor

*Note* $R^2_p$ = partial $R^2$; BF = Bayes factor; L = local climate variation; G = global temperature; P = parasite load; D = population density. $R^2_p$ values are computed from the different models’ $R^2$ values reported by Rouder and Morey (2012). Since these are rounded to four digits after the decimal point, results may differ slightly if the original data had been available for analysis. BFs were computed from $R^2_p$ values using one of our R functions (see Fig. 5) and the fact that *F* and $R^2_p$ stand in a one-to-one relationship. Default BFs are taken directly from Rouder and Morey’s paper

The Bayes factors for the effect-size priors were computed by means of calls to the R function shown in Fig. 5 for comparing $\mathcal M_2$ and $\mathcal M_\text {f}$ focused on large effects.

### ANOVA

**Fig. 6:**
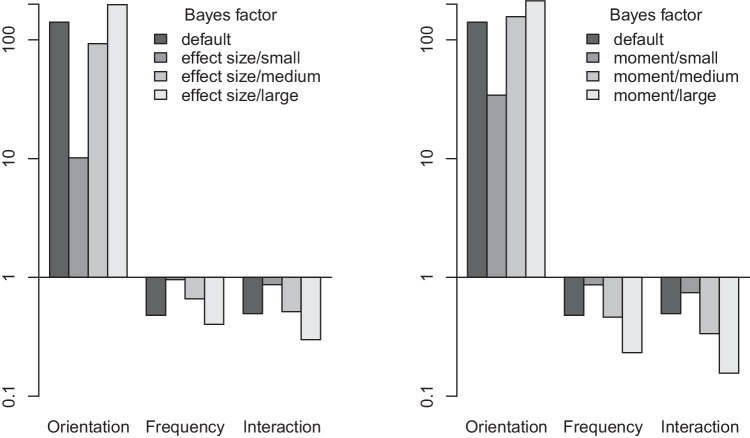
Default, effect-size (*left panel*) and moment (*right panel*) Bayes factors for hypothetical data by Rouder et al. (2012)

Rouder et al. (2012) presented a default Bayes factor analysis of hypothetical data from a 2 $\times$ 2 design with two between-participants factors and 40 fictive participants. The data are response times in seconds, between-participant factors are orientation (vertical vs. horizontal) and frequency (low vs. high) of Gabor gratings (see Rouder et al., 2012, Fig. 5, for the data). They were simulated so as to exhibit a large effect of orientation, but no effects of frequency and interaction. This is reflected both in the ANOVA table (see Table 9) as well as in the default, effect-size, and moment Bayes factors (see Fig. 6). As can be seen, the moment Bayes factors focusing on large effects best capture the underlying structure (large effect in orientation, null effects otherwise).

**Fig. 7:**
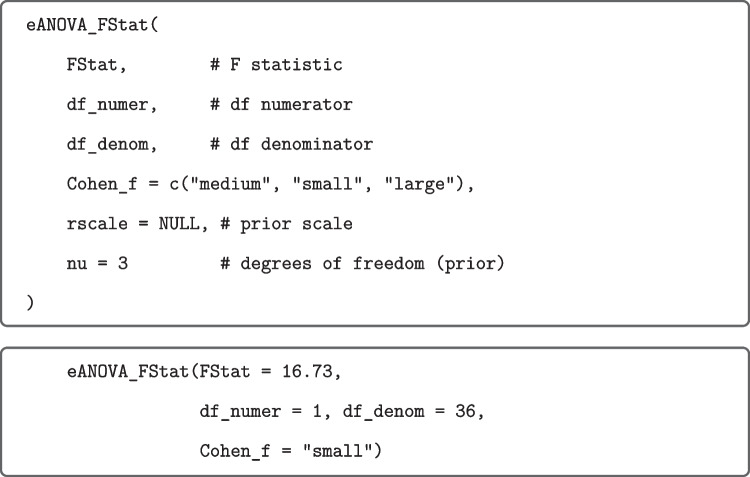
R Function eANOVA_FStat (*upper panel*) and application (*lower panel*) to hypothetical data from Rouder et al. (2012). *Note.* Argument Cohen_f corresponds to the prespecified effect size, with $f = 0.10$, $f = 0.25$, and $f = 0.40$ for small, medium, and large effects, respectively. If rscale and/or nu are not specified, they are set internally to the recommended values. The values of the moment Bayes factors can be computed by the similar function mANOVA_FStat. There are also R functions eANOVA_eta2p and mANOVA_eta2p proceeding from partial $\eta ^2$ values

The values of the effect-size Bayes factors were computed by means of calls to the R function eANOVA_FStat shown in Fig. 7 for the effect of orientation focused on small effects.

## Sensitivity and specificity

Relative to the default Bayes factors, how responsive are effect-size and moment Bayes factors in detecting real effects? Also, how efficient are they in accruing evidence for the null hypothesis when it is true?

**Fig. 8:**
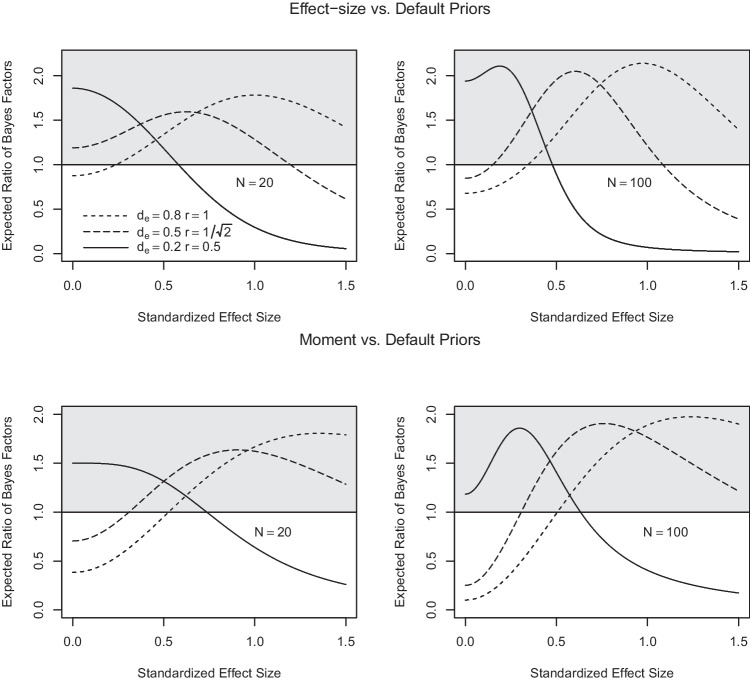
Expected ratio of Bayes factors $B_{10}$ of effect-size (*upper panels*) and moment Bayes factors (*lower panels*) relative to default Bayes factors as a function of true effect size. *Note.* *Left panels* show the ratios for samples of size $N=20$; *right panels* for $N=100$. The three lines correspond to ratios of the alternative (effect-size or moment) Bayes factors focusing on small, medium, and large effects versus default Bayes factors with, in order, small, medium, and wide scaling factors. The area with ratios larger than one is shaded

Figure 8 addresses the first question. It shows the expected ratio of the effect-size and moment Bayes factors and the default Bayes factor as a function of the underlying true effect size in the case of a one-sample *t* test for samples of size $N=20$ and $N = 100$. Values larger than one mean that the alternative (effect-size or moment) Bayes factor can be expected to be larger than the default Bayes factor by that factor. As can be seen, both effect-size and moment priors are more sensitive than the default Bayes factor across broad ranges of effect sizes and in particular in detecting effects in the vicinity of the targeted size. Effect-size Bayes factors perform somewhat better than moment Bayes factors for small to medium-sized true effects, whereas moment priors have the upper hand for larger effects.

**Table 9:** ANOVA table for hypothetical data by Rouder et al. (2012)

Effect	*df*	MSE	*F*	*p*	$\eta ^2_p$
Orientation	1, 36	0.01	16.73	<.001	0.32
Frequency	1, 36	0.01	1.04	.31	0.03
Interaction	1, 36	0.01	0.56	.46	0.02

*Note* *df* = degrees of freedom; MSE = mean squared error; *F* = *F* statistic, *p* = *p* value; $\eta ^2_p =$ partial eta-squared

**Fig. 9:**
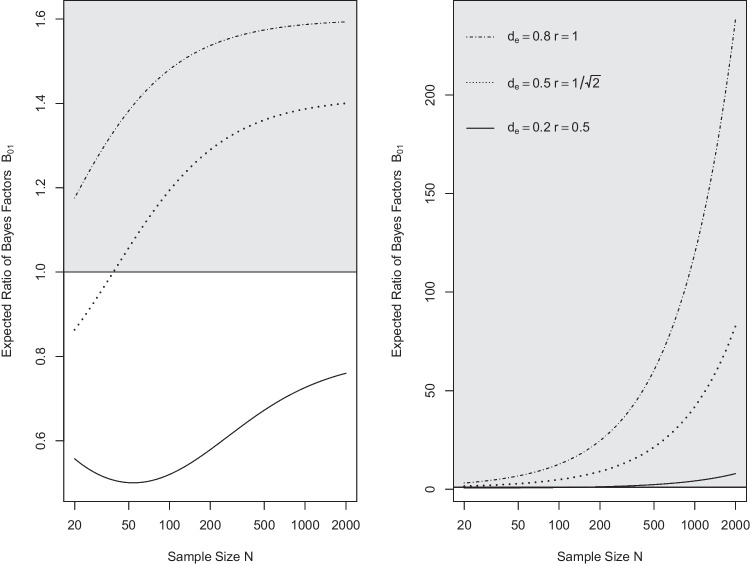
Expected ratio of Bayes factors $B_{01}$ of effect-size (*left panel*) and moment Bayes factors (*right panel*) relative to default Bayes factors as a function of sample size under $\mathcal M_0$. *Note.* The three lines correspond to ratios of the alternative (effect-size or moment) Bayes factors focusing on small, medium, and large effects versus default Bayes factors with, in order, small, medium, and wide scaling factors. The* horizontal axis* is on a logarithmic scale. The area with ratios larger than one is shaded

Figure 9 addresses the second question. It shows the expected ratio of the alternative (effect-size or moment) Bayes factors and the default Bayes factor for the preference for the null model over the alternative model (i.e., $B_{01}$) as a function of sample size. In this case, effect-size and moment Bayes factors turn out to show distinctly different profiles. The effect-size Bayes factor focusing on small effects is less efficient than the default Bayes factor in providing evidence for $H_0$ when this hypothesis is true, for the range of sample sizes covered. This reflects the fact discussed above that the effect-size priors become more similar to the prior in force under the null hypothesis as the prespecified effect size becomes small, compounded by the fact that extreme effect sizes are much less likely under the effect-size prior than under the default prior. Moderate gains can, however, be expected by using effect-size priors focused on medium and large effects.

In contrast, quite substantial gains can be expected from the use of moment priors focused on medium and large effects, especially for large sample sizes (see Fig. 9). But what causes this behavior? If the null hypothesis is true, then the data likelihood under $\mathcal M_1$ will become successively more concentrated on effects approaching zero as sample size increases. The moment priors, however, assign likelihoods approaching zero precisely for these effects. And thus, much of the data likelihood is suppressed by the moment prior, accounting for the comparatively large moment Bayes factors in favor of the null hypothesis. In consequence, if one’s idea of the alternative hypothesis includes the belief that the a-priori likelihood of effects vanishes the closer the effects are to zero, then the moment Bayes factor can be used to quickly accumulate evidence in favor of the null hypothesis as the sample size increases (see also Pramanik & Johnson, 2024). A formal argument given in the Supplementary Materials provides further insight into the underpinnings of this feature of moment priors (see Section “Large-sample consistency of moment Bayes factors”).

## Bayes factors and significance tests

Having introduced effect-size and moment Bayes factors, we will now shift gears, take a step back, and turn our attention to a more basic question: *How do hypothesis tests using Bayes factors relate to frequentist significance tests?*

The reason for pivoting this way so late in our discussion is that it will lead us to the insight that default, effect-size, and moment Bayes factors are in fact instances of *test-statistic-based Bayes factors*, originally proposed in a similar form by Johnson (2005). This insight has considerable practical implications, as it establishes the applicability of a simple method for computing Bayes factors to a wide range of hypothesis tests for which statistical power can be calculated.

### Differences between the Bayesian and classical approaches to hypothesis testing

Comparisons between Bayes factors and frequentist significance tests are made difficult by the fact that the two approaches serve different purposes. Bayes factors aim to quantify the evidence for one model relative to another model, whereas significance tests attempt to provide control over the error probabilities when deciding for or against $H_0$. That significance tests do not immediately provide a quantification of the evidence for a model representing the $H_0$ relative to a model representing the $H_1$ is therefore as true as it is unsurprising. As true and unsurprising as the fact that Bayes factors do not immediately provide control over the error probabilities in deciding for or against $H_0$ (but see Berger et al., 1997).

A perhaps more important difficulty is the fact that when the theories underlying Bayes factors and significance testing make reference to a “null hypothesis” and an “alternative hypothesis”, *they are referring to entirely different concepts*. In the case of significance testing, the null hypothesis $H_0:$ $\theta =0$, is a *simple* hypothesis, whereas the alternative $H_1:$ $\theta \ne 0$ is a *composite* hypothesis. A composite hypothesis is an (infinite) disjunction of simple hypotheses; that is, $\theta = \theta _x$ or $\theta = \theta _y$ or $\ldots$ for all values $\theta _x$, $\theta _y$, $\ldots$ different from zero. In other words, $H_1$ can be unpacked into an (infinitely large) collection of simple hypotheses, and the claim under $H_1$ is that one of the simple hypotheses in the collection is true. In contrast, for Bayes factors, the $H_1$ is represented by a model $\mathcal M_1$ that cannot be unpacked in any natural way. In this sense, the model $\mathcal M_1$ standing as a proxy for $H_1$ is more appropriately considered a simple hypothesis that is obtained when averaging across the parameter space weighted by the prior, than a composite hypothesis (for a similar point, see Tendeiro & Kiers, 2019).

Going back to significance testing, it presumes that $H_0$ and $H_1$ exhaust all the possibilities, such that either $H_0$ or $H_1$ must hold.^13^ This could not be more false for the Bayesian framework, as it is definitely *not* true that $\mathcal M_0$ and $\mathcal M_1$ exhaust the possibilities. Simply put, there are infinitely many alternative models defined by alternative priors that could be chosen to represent $H_1$ instead. And if there are parameters other than the tested one, namely $\theta$, then the same could be said of $H_0$. To bridge this gap, a Bayesian analysis would have to construct $H_1$ as the collection of models $\mathcal M$ with priors that* do not* concentrate all likelihood on the point $\theta = 0$, whereas $H_0$ would be the collection of models $\mathcal M$ with priors that *do* concentrate all likelihood on $\theta = 0$.

This unwieldy construction reveals the unhappiness of the marriage between Bayesian model selection and hypothesis testing. In this marriage, Bayesian model selection via the Bayes factor is portrayed as a (if not *the*) rational, normative approach to belief change in hypothesis testing. Once we assign positive prior probabilities to $H_0$ and $H_1$ with $P(H_0) + P(H_1)=1$, the Bayes factor then tells us how we should rationally change the prior probabilities in reaction to the data. And it may indeed make sense to assign positive prior probabilities to the null hypothesis that there is no effect and to the alternative hypothesis that there is an effect (e.g. Rouder et al., 2012; see also Gelman et al., 2013, Chap. 7.4).

But to make this Bayesian marriage work, we need to equate $P(H_1)$, our prior belief in the alternative hypothesis, with $P(\mathcal M_1)$, our prior belief in model $\mathcal M_1$. And this is where the proverbial “irreconcilable differences” make an appearance. $\mathcal {M}_1$ cannot have a positive prior probability assigned to it on the grounds that it represents $H_1$. After all, there is an infinity of models extremely similar to it that represent $H_1$ equally well. Because of this similarity, these models are to be assigned prior probabilities close if not equal to $P(\mathcal M_1)$. At the same time, there is the requirement for these very same probabilities to sum to a value smaller or equal to one – which is impossible to satisfy.

Note that we are not dealing with a practical issue here. The Bayes factor would yield practically equivalent support for all of these models – unless, of course, we have very large samples as we sometimes do in this age of big data. The issue at hand is one of *conceptual foundations*: If we want to appeal to the normative force of the laws of probability in order to ascribe a privileged role to statistical inference via Bayes factor, then it would probably be good to know what exactly these probabilities are probabilities of. Is my prior belief in $H_1$ the probability of a single model $\mathcal {M_1}$? As we just saw, this interpretation opens a can of worms. Maybe it is the prior probability of $\mathcal {M_1}$ conditional on both $\mathcal {M_0}$ and $\mathcal {M_1}$ or the probability of an equivalence or high-similarity class of models representing $H_1$? Such ideas raise a host of new problems. This manuscript is not the place to sort out the intricate questions surrounding subjective beliefs in substantive hypotheses, probabilities of models, and their relationship. For the current purposes, it is perhaps best to work around them. For instance, by speaking of Bayes factors as enabling quantitative comparisons between two models $\mathcal M_0$ and $\mathcal M_1$, each being one of usually infinitely many plausible representations of $H_0$ and $H_1$, respectively.

### Do significance tests allow one to quantify the evidence for or against the null?

Significance tests are often criticized as not quantifying the evidence for or against the $H_0$ appropriately. For example, the *p* value in Fisher’s approach to significance testing is often considered unfit to provide an appropriate assessment of this evidence (see, e.g., Wagenmakers, 2007; Wagenmakers, et al., 2017; Ly et al., 2020; Kruschke, 2010a, b; see also Huisman, 2023, for a recent summary of such criticisms). This is a justified criticism given that in Fisher’s framework, there is no alternative hypothesis $H_1$ being considered. But it is as justified as criticizing the Bayesian approach on the grounds that the marginal likelihood of the data under the model $\mathcal M_0$ representing the $H_0$ does not appropriately quantify the evidence for or against $\mathcal M_0$. What is needed in both cases is a specification of the $H_1$ or the alternative model $\mathcal M_1$ to provide a point of comparison. In significance testing, this leads to the Neyman-Pearson approach, which considers simple hypotheses $H_1:$ $\theta = \theta ^*$ with $\theta ^*$ being a fixed value other than zero. In the Neyman-Pearson tradition, we can fix $\alpha$ and $\beta$ error probabilities a priori and determine the sample size so that the error probabilities associated with the significance test are given by $\alpha$ and $\beta$.^14^ In the following, we review a number of methods on how to transform the outcomes of significance tests to Bayes factors.

The easiest case is to quantify the evidence implicit in the observation that the outcome of the significance test is significant or not. If this binary outcome is all that we retain from the data, then it is easy to see that a significant outcome implies a Bayes factor of $\frac{1-\beta }{\alpha }$ in favor of any $\mathcal M_1$ that concentrates all likelihood on $\theta = \theta ^*$ relative to any $\mathcal M_0$ that concentrates all likelihood on $\theta = 0$. For example, if $1-\beta$, the test power, is $80\%$, and $\alpha = 5\%$, a significant result implies a Bayes factor $B_{10}$ of no less than 16 in favor of $\mathcal M_1$. Conversely, the Bayes factor associated with a non-significant result is $\frac{\beta }{1-\alpha }$ so that a non-significant result implies a Bayes factor $B_{01} = 1/B_{10}$ in favor of the $H_0$ of 4.75 in this situation.^15^ For any model $\mathcal M_1$ with a prior $f(\theta )$ on the effect-size parameter, the Bayes factor for $\mathcal M_1$ relative to any model $\mathcal M_0$ that concentrates all likelihood on $\theta = 0$, is $B_{10} = \nicefrac {\int \Phi (\theta ) f(\theta ) \text {d}\theta }{\alpha }$ in the case of a significant test result, where $\Phi ()$ is the power function of the significance test. In the case of a non-significant test outcome, it is

$$ B_{10} = \nicefrac {(1 - \int \Phi (\theta ) f(\theta ) \text {d}\theta )}{(1 - \alpha )}. $$

What is the relationship of these Bayes factors to the default, effect-size, and moment Bayes factors discussed earlier on? In a nutshell, they are legitimate Bayes factors that do not, however, make full use of the observed data and the information therein as the default, effect-size, and moment Bayes factors do. And thus, if the default Bayes factor and/or the effect-size/moment Bayes factor are also computed, these Bayes factors may defeat the evidence provided by the Bayes factors based on only the binary outcome of the significance test just as a Bayes factor computed on one-half of the data may be defeated by the Bayes factor computed on the complete dataset.

### A Bayes factor based on *p* values

However, the significance test comes with more informative outcomes, in particular the much-maligned *p* value. In fact, if the *p* value is all that we retain from the data, then the Bayes factor contrasting $\mathcal {M}_1$ and $\mathcal M_0$ on the basis of this datum, the *p* value, is given by (e.g., Held & Ott, 2018):

$$\begin{aligned} B_{10} = \frac{\int g(p \mid \theta ) f(\theta ) \text {d}\theta }{ g(p \mid 0)} = \int g(p \mid \theta ) f(\theta ) \text {d}\theta , \nonumber \end{aligned}$$

where $g(p \mid \theta )$ is the density of the distribution of the *p* value given true effect $\theta$. Note that $g(p \mid 0)$ is 1, because the *p* value is uniformly distributed on the interval [0, 1] under $H_0$. This representation of the evidence contained in a *p* value is difficult to compute because expressions for the density $g(p \mid \theta )$ under the integral are usually not known. But where the test statistic, let us call it *t*, and its *p* value stand in a one-to-one relationship, we can make a change of variable from *p* to *t* in both numerator and denominator of the above equation. It follows that (e.g., Held & Ott, 2018):

5 $$\begin{aligned} B_{10} = \frac{\int f(t \mid \theta ) f(\theta ) \text {d}\theta }{f(t \mid 0)}, \end{aligned}$$

where $f(t \mid \theta )$ is the density of the test statistic given $\theta$.^16^

This density is the density of the distribution of the test statistic under the alternative hypothesis. For example, for the *t* tests this is the density of a noncentral *t* distribution with noncentrality parameter that is a known function of the effect parameter $\theta$; for the *F* test, it is the density of a noncentral *F* distribution with noncentrality parameter that is a known function (of the possibly vector-valued) effect parameter $\theta$. Like the default, effect-size, and moment Bayes factors, this *p* value-based Bayes factor depends on the data solely through the value of the test statistic.

But what is the relationship of the *p* value-based Bayes factor to the default, effect-size, and moment Bayes factors? Is it somehow worse or even better? Given that the default, effect-size and moment Bayes factors are based on the likelihoods of the original data rather than on only the summaries thereof provided by the test statistic, the expectation is that the former make more complete use of the information in the data than the latter. As Johnson (2008, p. 355) put it, “by reducing the information contained in the data to a single test statistic, some information is inevitably lost”.

To address this question, it is natural to compute the *p* value-based Bayes factors from Eq. 5 with the priors on the model parameters assumed for the default, effect-size, and moment Bayes factors. As shown in the Supplementary Materials, it turns out that for the testing cases considered here, the *p* value-based Bayes factor computed from Eq. 5 with these priors *is identical* to the default,^17^ effect-size, and moment Bayes factor. And so, contrary to expectations, *no information is actually lost*. Or to be more precise, whatever information is lost, it does not bear on the task of distinguishing between $\mathcal M_0$ and $\mathcal M_1$.

The equivalence of *p* value-based Bayes factors on the one hand default, effect-size, and moment Bayes factors on the other is surprising given that they have been reached by very different routes. Default, effect-size, and moment Bayes factors depart from the raw data; the fact that they ultimately depend on the data only via the summary provided by the test statistic is an emergent property reached in a complicated derivation. They also involve more assumptions than the *p* value-based Bayes factors since the latter do not need any distributional assumptions about parameters outside the hypotheses tested that are shared by the two models contrasted. And so, how is it possible that both kinds of Bayes factors are identical? We explore this question in the Supplementary Materials in which we argue that the *p* value-based Bayes factor will in general be similar to a Bayes factor computed from a full set of distributional assumptions on all model parameters when vague priors are used for the parameters shared by both models. Taken together, these considerations suggest an approach to hypothesis testing by Bayes factors for many more hypothesis tests than covered so far.

## A Bayes factor based on the test statistic

The equivalence between the two kinds of Bayes factors creates an opportunity: to compute a Bayes factor for a given hypothesis testing problem, one can capitalize on the classical frequentist solution for it. For most established significance tests, the density of the distribution of the test statistic *T* from the classical frequentist significance test is known for all parameter values $\theta$ involved in the tested hypotheses. This makes it possible to compute the Bayes factor based on the test statistic Eq. (5).

One difficulty is, however, that if $\theta$ is vector-valued this still implies a computationally costly multidimensional integration. However, for the significance tests discussed by Cohen (1988) and treated in G*Power 3 (Faul et al., 2007), the density of the test statistic in most cases depends on the parameter $\theta$ only through a one-dimensional parameter, namely the effect size (e.g., Johnson, 2005, 2008).

It follows that we can parameterize the density of the test statistic in terms of the effect-size parameter and need to integrate only over the prior implied for it by the prior distribution assumed for $\theta$.^18^

This is, however, a unidimensional integration. If $\xi$ is the appropriate effect-size measure and *t* the observed value $t = T(\varvec{y})$ of the test statistic, we have:

6 $$\begin{aligned} B_{10} = \frac{\int f( t \mid \xi ) f(\xi ) \text {d}\xi }{f (t \mid \xi = 0)}, \end{aligned}$$

where $f(\xi )$ is the prior distribution of effect sizes as implied by the prior assumptions about standardized effects.

The prior distribution of the effect-size parameter can be trivial to determine. For example, for the effect-size Bayes factor for *t* tests, the prior for Cohen’s *d* is simply a mixture of two shifted and scaled *t* distributions. In other cases, however, the relevant prior is much more difficult to derive. For example, the prior implied for Cohen’s effect-size measure *f* in regression and ANOVA models is difficult to derive and does not correspond to any well-known family of distributions (see Supplementary Materials).

Fortunately, given the subjective nature of all of these priors, all that we need to compute the Bayes factor based on the test statistic via Eq. 6 is, however, to specify a *reasonable* prior distribution on the effect-size measure associated with the test in question. It need not be the one that is implied by explicit assumptions about the prior distribution of possibly multidimensional model parameters in the sense explained in Footnote 18 as long as it is a sensible one consistent with the effect sizes that are to be expected in the field of inquiry and as long as it meets with acceptance by a wider circle of researchers.

**Fig. 10:**
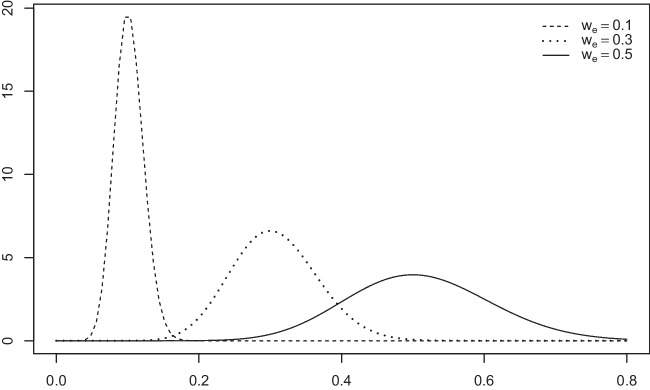
Prior distribution of effect size *w* focused on $w_e$ induced by gamma priors on $w^2$

For example, consider the chi-squared goodness-of-fit tests or model comparisons that arise frequently in categorical data analyses (Bishop et al., 1975; Agresti, 2002), such as the analysis of contingency tables via log-linear models or multinomial processing tree models (Batchelder & Riefer, 1999), among many others. Here, the test statistic is most commonly $G^2$, the log-likelihood ratio statistic comparing a reduced and a full model or, asymptotically equivalently, Pearson’s chi-squared statistic $\chi ^2$. The effect-size measure is Cohen’s *w* with the convention that ‘small’, ‘medium’, and ‘large’ effects are demarcated by, in order, $w = .10$, $w =.30$, and $w = .50$. For large datasets, the distribution of the test statistic under the alternative hypothesis is a noncentral $\chi ^2-$distribution that relates to *w* via the noncentrality parameter $ncp = N w^2$, where *N* is the sample size. The formula for computing $w^2$ resembles the computation of a chi-squared statistic (Cohen, 1988, Chap. 7), and the chi-squared distribution is a member of the family of Gamma distributions. Hence, it seemed reasonable to choose a Gamma distribution as prior for $w^2$. We chose the parameter values of the Gamma priors so that the priors induced thereby for *w* are focused on effect size $w_e$ with a standard deviation of $0.2w_e$ (see Fig. 10).^19^

Assuming that one considers these priors reasonable effect-size priors, and if *x* is the observed value of the test statistic $G^2$ for a model comparison with $\nu$ degrees of freedom, the Bayes factor would be

$$\begin{aligned} B_{10} \!=\! \frac{\int f( x \mid \nu , N w^2 ) f_\gamma (w^2 \mid \textrm{shape} \!=\! 6.6, \textrm{rate} \!=\! (2*6.6-1)/w_e^2) \text {d}w^{2}}{f (x \mid v, 0)}, \end{aligned}$$

where $f( x \mid \nu , N w^2 )$ is the density of the noncentral $\chi ^2$ distribution with $\nu$ degrees of freedom and noncentrality parameter $N w^2$.

**Fig. 11:**
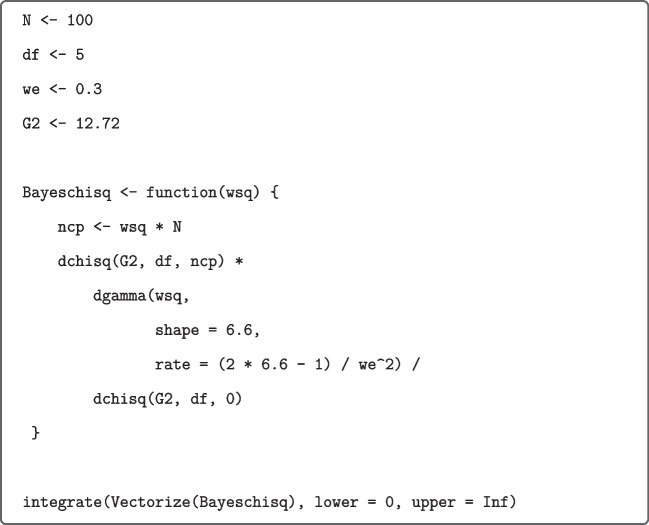
Computing the Bayes factor based on the chi-squared statistic $G^2$

These densities are readily available as R functions as are the densities of a wide range of obvious choices for effect-size priors. For example, for a $G^2$ value of 12.72 with five degrees of freedom and $w_e = 0.3$ based on $N=100$ observations, we can compute the test-statistic-based Bayes factor in R by means of a few lines of code as shown in Fig. 11. This computation yields a Bayes factor $B_{10}$ of 5.14.

Taken together, the test-statistic-based Bayes factor constitutes a simple procedure for computing Bayes factors for many hypothesis testing problems for which an effect-size measure is specified, and the distribution of the test statistic as a function of the effect size under the alternative hypothesis is known. The procedure involves specifying a reasonable prior on the effect-size measure based on experience, intuition, and consensus-building efforts in the community of researchers in the field, and integrating, using this prior, over the density of the test statistic.

## Discussion

Default Bayes factors have desirable theoretical and practical advantages. On the theoretical side, they exhibit a number of consistency and invariance properties that reasonable priors should have: They show scale invariance, large-sample consistency, and consistency in information. On the practical side, they are relatively easy to compute and their application has been much facilitated by accessible software. However, as acknowledged by Rouder and Morey (2012), they are subjective priors, implying that different researchers may find alternative priors more plausible or useful. Alternative priors have, however, rarely been explored, one obstacle being that Bayes factors are generally difficult to compute.

Given that Bayes factors strongly depend on the priors adopted, their choice requires careful justification. Two features of the default priors motivated the development of alternatives: Even though the default priors are meant to represent the alternative hypothesis, their single most likely value is the null effect, and effect sizes are assumed to be more likely the closer they are to a null effect. At the same time, unreasonably large effect sizes still receive substantial likelihood.

To remedy these shortcomings, we discussed two families of alternative priors, effect-size priors and moment priors, for the most common analyses encountered in psychological research: for one-sample and two-sample *t* tests, for ANOVA, and for regression analyses. These priors share the desirable theoretical properties exhibited by the default priors and are as easy to compute as the default priors. We provide an R package to perform the necessary computations. The alternative (effect-size or moment) priors with the recommended settings have considerably less heavy tails than the default priors, rendering very large effect sizes unlikely. Moreover, they are focused on effects of a size that can be set by the researcher. In consequence, effects of the prespecified magnitude rather than null effects are assumed to be most likely.

Unlike the default Bayes factors as implemented in the R package BayesFactor (Morey and Rouder, 2013) and the standalone software JASP (Love et al., 2019); Wagenmakers et al., 2018), the effect-size and moment Bayes factors proposed here satisfy *framing invariance*, meaning that using different, but equivalent approaches to test the same hypotheses (e.g., via ANOVA analyses versus via regression analyses) yield the same Bayes factors. Unlike Gronau et al. ’s (2020) informed priors for *t* tests, the effect-size and moment priors satisfy *predictive matching*, meaning that the Bayes factor for uninformative data does not suggest evidence in favor of $H_0$ or $H_1$ (i.e., its value is 1). And unlike Pramanik and Johnson ’s (2024) nonlocal alternative priors for *t* tests, they satisfy *consistency in information*, meaning that the preference for the model representing the alternative hypothesis increases without bounds as the test statistic (*t* or *F* as the case may be) tends to infinity.

The effect-size and moment priors thereby expand the researchers’ menu of choices of priors that satisfy Jeffreys ’s (1942) desiderata for prior choice. We expect them to be particularly useful for researchers who share our intuitions regarding the relative plausibility of the default priors and the effect-size and moment priors. However, even researchers preferring the default priors can make good use of these new techniques for the purpose of studying the robustness of Bayes factor-based inferences vis-à-vis different choices of priors. This is all the more relevant as robustness studies are being regularly demanded in the Bayesian literature (e.g., Liseo, 2000), but rarely conducted. Lastly, there are also growing concerns regarding the uncertainty surrounding effect sizes (e.g., Davis-Stober et al., 2022; Pek et al., in press) – the present families of priors provide researchers with new tools for better expressing that uncertainty.

One interesting alternative to the Bayes factors considered so far is given by the fractional Bayes factor (O’Hagan, 1995, 1997). Although “not a genuine Bayes factor” (O’Hagan, 1995, p. 117), the fractional Bayes factor has many of the consistency and coherence properties of Bayes factors (O’Hagan, 1997). Mulder (2014) has proposed an adjusted version of it, and Mulder et al. (2021) develop the fractional and the adjusted fractional Bayes factors for many of the testing problems considered here and additional ones via their R package BFpack. One attractive feature of these Bayes factors as implemented in BFpack is that they are computed in a fully data-driven way: The data are used to estimate (implicit) priors as well as to discriminate between the hypotheses under scrutiny. When computed for the above example applications, they performed similarly as the default Bayes factors with the exception of the ANOVA problem, in which they were mostly more in favor of the null hypothesis than the other Bayes factors (see the Supplementary Materials for their values). It would be interesting to compare the Bayes factors considered here and fractional and adjusted fractional Bayes factors systematically in terms of theoretical consistency properties as well as in terms of sensitivity and specificity in future work.

Juxtaposing hypothesis tests by significance tests and hypothesis tests by Bayes factors, we pointed out that their comparison is difficult due to the fact that they serve different purposes: The control of error probabilities *versus* the quantification of evidence for a model $\mathcal M_1$ representing the alternative hypothesis relative to a model $\mathcal M_0$ representing the null hypothesis. Moreover, equating $H_0$ with $\mathcal M_0$ and $H_1$ with $\mathcal M_1$ leads to a number of conceptual difficulties that rob the Bayesian approach of much of its normative appeal as a rational approach to testing between $H_0$ and $H_1$. This train of thought raised the question of how to transform the outcomes of significance tests onto the Bayes factor metric, leading to the novel insight that default, effect-size, and moment Bayes factors are all instances of test-statistic-based Bayes factors (Johnson, 2005, 2008, but see Footnote 17). The test-statistic-based Bayes factor is a useful approach to Bayes factor-based hypothesis tests that allows one to compute valid Bayes factors for most hypothesis testing problems for which an effect-size measure has been proposed and test power can be computed. This approach has the potential to lead to a software program for conducting Bayes factor analyses of similar scope as the famous G*Power 3 program (Faul et al., 2007).

The test-statistic based Bayes factor will also be useful for researchers who prefer other priors than the effect-size and moment priors. Even those researchers may find the alternative (effect-size or moment) priors useful for fast and convenient robustness studies as frequently considered necessary in the Bayesian literature. Despite their desirable theoretical and practical properties, it is nevertheless important to acknowledge that the alternative priors are no less subjective than the default priors. Like for all subjective priors, adopting any of them will require good arguments and hard work on building consensus among experts in the field (Rouder et al., 2016), perhaps in the form of Delphi studies (Taylor, 2020) for each field of application. For most research purposes, a viable alternative is, of course, to conduct the appropriate significance test.

## Supplementary Information

Below is the link to the electronic supplementary material.

Supplementary file 1 (pdf 443 KB)

## Data Availability

We do not report new data.

## Footnotes

1 Whereas the present manuscript focuses on Bayesian hypothesis testing via Bayes factors, we note that another principled approach to Bayesian hypothesis testing relies on the posterior distribution of parameters (e.g., Kruschke, 2015, chap. 12). A discussion of the relative merits of both approaches is beyond the scope of this manuscript (see, e.g., Tendeiro & Kiers, 2019; van Ravenwzaaij & Wagenmakers, 2022). Moreover, the exposition will focus on non-directional tests of strict null hypotheses (i.e., $H_0: \theta = 0$), but the methods developed here are easily applied to test directional alternative hypotheses as well as to test interval hypotheses specified as regions of effects smaller (or larger) than a predetermined size (e.g., in terms of regions of practical equivalence, Kruschke, 2015, chap. 12) as well as to test simple (point) hypotheses specified in terms of fixed values $\theta _0$ other than zero (i.e., $H_0: \theta = \theta _0$); see Supplementary Materials.

2 Note, however, that there is no simple relation between effect sizes and the scale factors of default priors for ANOVA and regression problems.

3 This reflects the fact that $\mathcal M_0$ and $\mathcal M_1$ can be seen to differ only in the prior assumed for *d*, the prior for $\mathcal M_0$ focusing all probability mass on $d=0$; the prior for $\mathcal M_1$ distributing probability across the range of possible values of *d* (see Table 2).

4 Note, however, that JASP (Love et al., 2019) offers the option to specify informed priors for *t* tests and that Mulder et al. (2021) have developed an R package, BFpack, that implements so-called fractional Bayes factors and variations thereof as elaborated on in the General discussion.

5 For the *t* test problems considered first, this goal is automatically satisfied if the prior on the effect parameter *d* is symmetric around zero.

6 For the sake of readability, the main text of this paper will focus on two-sided tests. Where applicable, one-sided tests are, however, made available in the accompanying R package.

7 Note that different standardizations of $f^2$ (and *f*, which will be used as effect size in the context of ANOVA) can be found in the literature. We prefer to standardize by the effective sample size $N-p$ in order to have unit priors (see below), which is similar to Cohen (1988, p. 414) who proposes to standardize by $N-p+1$. A case can also be made for standardizing by *N*, which is mostly used by G*Power 3 (Faul et al., 2007). If this standardization is preferred, users should enter $f^2 \nicefrac {N}{N-p}$ for $f^2$ (and $f \sqrt{\nicefrac {N}{N-p}}$ for *f*) in our R package. Further variations exist in the context of ANOVAs with repeated measures; see Supplementary Materials, section “Coding ANOVA Models as Regression Models” for additional discussion.

8 For ANOVA Bayes factors that also only require one-dimensional integration, see Wetzels et al. (2012)

9 Rouder et al. (2012) point to a potential problem with large-sample consistency for ANOVA designs involving within-subject factors. The issue is that increases in *N* imply increases in the number of participants and therefore increases in the dimensionality of the reduced model, with each person having an effect parameter. As discussed in the Supplementary Materials, such ANOVA designs can, however, be coded as regression models of fixed dimensionality, that is without person parameters, so that the problem does not arise for the current Bayes factors.

10 Johnson and Rossell (2010, p. 155) state without proof that consistency in information is obtained whenever $N> p + q + \nu -2$.

11 These adjustments also have to be made for the scale factor *r* if it is not set according to the recommendations spelled out below.

12 Note, however, that this recommendation is based on the necessarily limited range of setups that we explored.

13 Provided the assumptions about how the data are distributed are granted as true.

14 Also note that $\beta$ provides an upper bound of the probability of erroneously deciding for $H_0$ for values $\theta$ more extreme than $\theta ^*$.

15 For more extreme values of $\theta$ than $\theta ^*$, these Bayes factors increase in size.

16 For some one-dimensional two-sided tests such as the *t* test, different values of the test statistic such as $t=a$ and $t=-a$ are associated with the same *p* value, so that *p* and *t* are not in a one-to-one relationship. In many such cases, including the current Bayes factors, the Bayes factor based on the test statistic nevertheless equals the *p* value-based Bayes factor for symmetry reasons, and for the sake of simplicity, we will treat both Bayes factors as equivalent for current purposes.

17 Note that this holds for all default Bayes factors other than the ANOVA ones.

18 What does it mean for a prior distribution to be implied by other priors? Consider as an example, Cohen’s effect-size parameter $f^2$ for regression problems. It is not itself a model parameter, but it is defined as a function of the model parameters, for which prior distributions are specified. Its prior distribution can therefore be computed from the prior distributions of the model parameters that define it and in this sense, its prior distribution is implied by these other parameters’ priors.

19 According to Cohen (1988), “it is not generally necessary to make provision for $\varvec{w}$ [*w*] greater than .90” (p.218), justifying the use of relatively small standard deviations.

## Code Availability

The R package and source code for computing the alternative Bayes factors is available at https://github.com/meyer-grant/emBayesFactor.

## Declarations

### Open Practices Statement

We do not report new data. The R package for computing the alternative Bayes factors is available at https://github.com/meyer-grant/emBayesFactor.
