# Correction: On Bayes factors for hypothesis tests

**DOI:** 10.3758/s13423-025-02645-1

**Published:** 2025-02-10

**Authors:** Karl Christoph Klauer, Constantin G. Meyer-Grant, David Kellen

**Affiliations:** 1 Department of Psychology, Albert-Ludwigs-Universität Freiburg, 79085 Freiburg, Germany; 2 Department of Psychology, Syracuse University, Syracuse, NY USA

**Correction: Psychonomic Bulletin & Review**

10.3758/s13423-024-02612-2

Due to an error by the publisher, the paper was published with an incorrect Fig. 2. The correct figure is shown here.

**Fig. 2:**
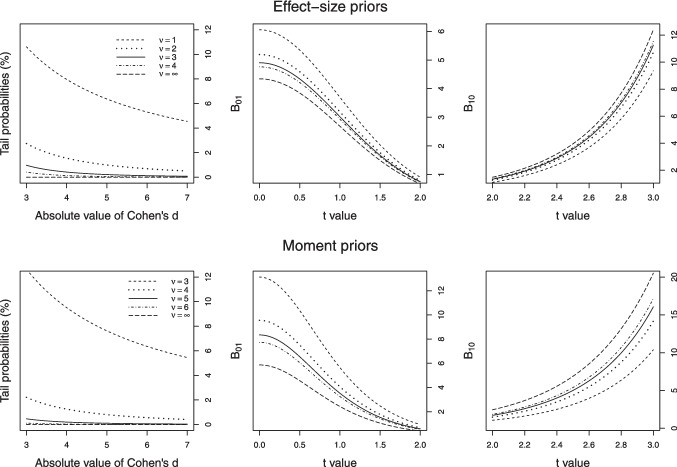
Tail probabilities and Bayes factor values for effect-size and moment priors for different degrees of freedom ν. *Note.* Complementary cumulative probabilities (*left panel*) of effect sizes, values of B_01_ (*middle panel*) for t-values between 0 and 2 and B_10_ (*right panel*) for t-values between 2 and 3 under effect-size priors with *d*_*e*_ = 0.3 and *r* = 0.5 (*upper panels*) and moment priors with *d*_*e*_ = 0.3 (*lower panels*) for different degrees of freedom ν

