# Supplementary material for: On Bayes factors for hypothesis tests
Source: Psychon Bull Rev. 2024 Nov 25;32(3):1070–94. doi: 10.3758/s13423-024-02612-2 (PMC12092502; doi:10.3758/s13423-024-02612-2)
Supplement: Supplementary file 1 — (pdf 443 KB) [file 13423_2024_2612_MOESM1_ESM.pdf]

Supplementary Materials

A.1 Bayes Factors for t Tests

Expressed in the present notation, Gronau et al. (2020) show that the Bayes factor for a one-sample t test with a shifted and scaled t prior is given by:

$$B_{10} = \int_{-\infty}^{\infty} \frac{f_{nct}(t \mid N-1, \sqrt{N}d)}{f_{nct}(t \mid N-1, 0)} f_t(d \mid \nu, d_e, r)\ \mathrm{d}d,$$

where $f_{nct}(t \mid N-1, x)$ is the density of the noncentral t distribution with $N-1$ degrees of freedom and noncentrality parameter x. From here, it is easy to derive the formula from Table 4 in the body of the paper. The proof for the two-sample t test proceeds analogously. The Bayes factors for moment priors can also be derived following the proofs provided by Gronau et al. (2020, supplemental material) analogously.

A.2 Bayes Factors in Multiple Linear Regression

A.2.1 Data Likelihood with Ancillary Parameters Integrated Out

Let $M = N - p$, $\Omega = \Sigma/M$, and $\boldsymbol{\gamma}$ be the standardized effects, $\boldsymbol{\gamma} = \Omega^{\frac{1}{2}}\boldsymbol{\beta}_2$, using the unique square root of the positive definite matrix Ω. Note that the effect size $\lambda^2 = \boldsymbol{\gamma}^t\boldsymbol{\gamma}$ and let $\boldsymbol{\gamma} = \lambda\boldsymbol{\gamma}'$. We begin by showing that

$$\int_E \left(\iint f(\boldsymbol{y} \mid \boldsymbol{\beta}_1, \Omega^{-\frac{1}{2}}\boldsymbol{\gamma}, \epsilon) f(\boldsymbol{\beta}_1) f(\epsilon)\, \mathrm{d}\boldsymbol{\beta}_1\, \mathrm{d}\epsilon \right) \mathrm{d}\mathcal{B}(\boldsymbol{\gamma}') = C f_F(x \mid q, M-q, M\lambda^2), \tag{A1}$$

where

- $f(\boldsymbol{y} \mid \boldsymbol{\beta}_1, \Omega^{-\frac{1}{2}}\boldsymbol{\gamma}, \epsilon)$ is the density of the distribution of the data $\boldsymbol{y}$ as defined in Table 6 in the body of the paper with $\boldsymbol{\beta}_2$ replaced by $\Omega^{-\frac{1}{2}}\boldsymbol{\gamma}$ and σ^2 by the precision $\epsilon = 1/\sigma^2$,
- x is the value of the F statistic for testing the reduced model against the full model,
- $f_F(x \mid q, M-q, M\lambda^2)$ is the density of the noncentral F distribution with q and $M-q$ degrees of freedom and noncentrality parameter $M\lambda^2$,
- C is a factor that depends only on the data $\boldsymbol{y}$ and the design matrix X_1, and

- $\mathcal{B}$ is the probability measure on the unit sphere that assigns equal likelihood to each point $\boldsymbol{\gamma}'$ with $||\boldsymbol{\gamma}'|| = 1$, and E is the set of points on the unit sphere. It defines a uniform distribution of effects of size $\lambda^2 = 1$.

Note that

$$f(\boldsymbol{y} \mid \boldsymbol{\beta}_1, \Omega^{-\frac{1}{2}}\boldsymbol{\gamma}, \epsilon) = \left(\frac{\epsilon}{2\pi}\right)^{(N/2)} e^{-\frac{1}{2}\epsilon||\boldsymbol{y}-X_1\boldsymbol{\beta}_1-\epsilon^{-\frac{1}{2}}X_2\boldsymbol{\beta}_2||^2}$$

and that

$$||\boldsymbol{y} - X_1\boldsymbol{\beta}_1 - \epsilon^{-\frac{1}{2}}X_2\boldsymbol{\beta}_2||^2 = (\boldsymbol{\beta}_1 - \hat{\boldsymbol{\beta}}_1)^t X_1^t X_1(\boldsymbol{\beta}_1 - \hat{\boldsymbol{\beta}}_1) - \hat{\boldsymbol{\beta}}_1^t X_1^t X_1 \hat{\boldsymbol{\beta}}_1 + A,$$

where

$$\begin{aligned} \hat{\boldsymbol{\beta}}_1 &= \left(X_1^t X_1\right)^{-1} X_1^t(\boldsymbol{y} - \epsilon^{-\frac{1}{2}}X_2\boldsymbol{\beta}_2), \text{ and} \\ A &= \boldsymbol{y}^t\boldsymbol{y} + \epsilon^{-1}\boldsymbol{\beta}_2^t X_2^t X_2\boldsymbol{\beta}_2 - 2\epsilon^{-\frac{1}{2}}\boldsymbol{\beta}_2^t X_2^t\boldsymbol{y}. \end{aligned}$$

Hence, with $f(\boldsymbol{\beta}_1) = 1$

$$\int f(\boldsymbol{y} \mid \boldsymbol{\beta}, \epsilon) f(\boldsymbol{\beta}_1)\, \mathrm{d}\boldsymbol{\beta}_1 = \left(\frac{\epsilon}{2\pi}\right)^{\frac{M}{2}} |X_1^t X_1|^{-\frac{1}{2}} e^{-\frac{1}{2}\epsilon(A - \hat{\boldsymbol{\beta}}_1^t X_1^t X_1 \hat{\boldsymbol{\beta}}_1)}.$$

Let

$$\begin{aligned} P_{\text{red}} &= X_1(X_1^t X_1)^{-1}X_1^t, \text{ and} \\ P_{partial} &= (I - P_{\text{red}})X_2\Sigma^{-1}X_2^t(I - P_{\text{red}}), \end{aligned}$$

and note that

$$\epsilon(A - \hat{\boldsymbol{\beta}}_1^t X_1^t X_1\hat{\boldsymbol{\beta}}_1) = \epsilon\boldsymbol{y}^t(I - P_{\text{red}})\boldsymbol{y} + M\lambda^2 - 2\epsilon^{\frac{1}{2}}\sqrt{M}\boldsymbol{\gamma}^t\boldsymbol{\gamma}^*$$

with $\boldsymbol{\gamma}^* = \Sigma^{-\frac{1}{2}}X_2^t(I - P_{\text{red}})\boldsymbol{y}$.

We can write $\sqrt{M}\boldsymbol{\gamma}^t\boldsymbol{\gamma}^*$ as

$$\sqrt{M}\boldsymbol{\gamma}^t\boldsymbol{\gamma}^* = \sqrt{M\lambda^2}\sqrt{\boldsymbol{y}^t P_{partial}\boldsymbol{y}}\,(\boldsymbol{\gamma}')^t(\frac{1}{||\boldsymbol{\gamma}^*||}\boldsymbol{\gamma}^*).$$

The term $(\boldsymbol{\gamma}')^t(\frac{1}{||\boldsymbol{\gamma}^*||}\boldsymbol{\gamma}^*)$ is the inner product of $\boldsymbol{\gamma}'$ with another vector of length 1. Because the uniform distribution on the unit sphere is rotation-invariant, we can choose this other vector to be $\boldsymbol{a} = (1, 0, \ldots, 0)^t$ without changing the distribution of the inner product over the unit sphere. The uniform distribution of $\boldsymbol{\gamma}'$ on the unit sphere can be obtained as the distribution

of $\boldsymbol{z}/||\boldsymbol{z}||$ with $\boldsymbol{z} = (z_1, \ldots, z_q)$ and the z_i following independent standard normal distributions. Thus, the inner product follows the same distribution as the variable $\phi = 1/||\boldsymbol{z}||\boldsymbol{z}^t\boldsymbol{a} = z_1/||\boldsymbol{z}||$.

From here, it is relatively easy to show that the density of the distribution of ϕ is for $q > 1$

$$f(\phi) = \frac{\Gamma(\frac{q}{2})}{\sqrt{\pi}\Gamma(\frac{q-1}{2})}(1-\phi^2)^{\frac{q-1}{2}-1}. \tag{A2}$$

Because the function under the integral over $\mathcal{B}(\boldsymbol{\gamma}')$ depends on $\boldsymbol{\gamma}'$ only via the inner product, we can replace the inner product by ϕ and integration over $\mathcal{B}(\boldsymbol{\gamma}')$ by integration over ϕ using the density $f(\phi)$.

Hence with $f(\epsilon) = 1/\epsilon$,

$$\int_E \left(\iint f(\boldsymbol{y} \mid \boldsymbol{\beta}_1, \Omega^{-\frac{1}{2}}\boldsymbol{\gamma}, \epsilon) f(\boldsymbol{\beta}_1) f(\epsilon)\, \mathrm{d}\boldsymbol{\beta}_1\, \mathrm{d}\epsilon \right) \mathrm{d}\mathcal{B}(\boldsymbol{\gamma}') =$$
$$\int_{-1}^{1} \int \left(\frac{\epsilon}{2\pi}\right)^{\frac{M}{2}} \frac{1}{\epsilon} |X_1^t X_1|^{-\frac{1}{2}} e^{-\frac{1}{2}M\lambda^2} e^{-\frac{1}{2}\epsilon \boldsymbol{y}^t(I-P_{\text{red}})\boldsymbol{y} + \epsilon^{\frac{1}{2}}\sqrt{M\lambda^2}\sqrt{\boldsymbol{y}^t P_{partial}\boldsymbol{y}}\ \phi}$$
$$\frac{\Gamma(\frac{q}{2})}{\sqrt{\pi}\Gamma(\frac{q-1}{2})}(1-\phi^2)^{\frac{q-1}{2}-1}\, \mathrm{d}\epsilon\, \mathrm{d}\phi.$$

Note that

$$\iint \frac{\Gamma(\frac{q}{2})}{\sqrt{\pi}\Gamma(\frac{q-1}{2})}(1-\phi^2)^{\frac{q-1}{2}-1} \omega^{M-1} e^{-\omega^2+\rho\omega\phi}\, \mathrm{d}\omega\, \mathrm{d}\phi = \frac{1}{2}\Gamma(\frac{M}{2})\, {}_1F_1(\frac{M}{2}; \frac{q}{2}; \frac{\rho^2}{4}),$$

where ${}_1F_1(\cdot;\cdot;\cdot)$ is the confluent hypergeometric function of the first kind (Abramowitz & Stegun, 1972, Chap. 15) and that the partial coefficient of determination R^2_{partial} equals $\boldsymbol{y}^t P_{\text{partial}}\boldsymbol{y}/\boldsymbol{y}^t(I-P_{\text{red}})\boldsymbol{y}$.[1] It follows with a change of variable $\epsilon = \omega^2/\alpha$ ($\mathrm{d}\epsilon = 2\omega/\alpha\, \mathrm{d}\omega$ with $\alpha = \frac{1}{2}\boldsymbol{y}^t(I-P_{\text{red}})\boldsymbol{y}$):

$$\int_E \left(\iint f(\boldsymbol{y} \mid \boldsymbol{\beta}_1, \Omega^{-\frac{1}{2}}\boldsymbol{\gamma}, \epsilon) f(\boldsymbol{\beta}_1) f(\epsilon)\, \mathrm{d}\boldsymbol{\beta}_1\, \mathrm{d}\epsilon \right) \mathrm{d}\mathcal{B}(\boldsymbol{\gamma}') =$$
$$(\pi \boldsymbol{y}^t(I-P_{\text{red}})\boldsymbol{y})^{-\frac{M}{2}} \Gamma(\frac{M}{2}) |X_1^t X_1|^{-\frac{1}{2}} e^{-\frac{1}{2}M\lambda^2}\, {}_1F_1(\frac{M}{2}; \frac{q}{2}; \frac{1}{2}M\lambda^2 R^2_{partial}) =$$
$$C' e^{-\frac{1}{2}M\lambda^2}\, {}_1F_1(\frac{M}{2}; \frac{q}{2}; \frac{1}{2}M\lambda^2 R^2_{partial}), \tag{A3}$$

where C' depends only on the data and design matrix X_1.

[1] If an intercept is not part of the reduced model, this quantity is usually not called the partical coefficient of determination, but this is irrelevant for our derivation.

If x is the value of the F statistic, then

$$\frac{1}{2}M\lambda^2 R^2_{\text{partial}} = \frac{1}{2}M\lambda^2 \frac{qx}{M-q+qx}.$$

Hence,

$$\int_E \left(\iint f(\boldsymbol{y} \mid \boldsymbol{\beta}_1, \Omega^{-\frac{1}{2}}\boldsymbol{\gamma}, \epsilon) f(\boldsymbol{\beta}_1) f(\epsilon)\, \mathrm{d}\boldsymbol{\beta}_1\, \mathrm{d}\epsilon \right) \mathrm{d}\mathcal{B}(\boldsymbol{\gamma}') = C f_F(x \mid q, M-q, M\lambda^2). \tag{A4}$$

A.2.2 Prior Induced by Effect-Size Priors on the Effect-Size Parameter

The marginal likelihood $l_1(\boldsymbol{y} \mid \mathcal{M}_0)$ is given by

$$\int \left(\iint f(\boldsymbol{y} \mid \boldsymbol{\beta}_1, \boldsymbol{\beta}_2, \epsilon) f(\epsilon)\, \mathrm{d}\boldsymbol{\beta}_1\, \mathrm{d}\epsilon \right) \left(\int_{E_{\Omega,f}} f_t(\boldsymbol{\beta}_2 \mid \nu, \boldsymbol{\mu}, r^2\Omega^{-1})\, \mathrm{d}\sigma_{\Omega,f}(\boldsymbol{\mu}) \right) \mathrm{d}\boldsymbol{\beta}_2,$$

where $f_t(\boldsymbol{\beta}_2 \mid \nu, \boldsymbol{\mu}, r^2\Omega^{-1})$ is the density of the multivariate t distribution with ν degrees of freedom, mean $\boldsymbol{\mu}$, and scale matrix $r^2(\Omega)^{-1}$, while $\sigma_{\Omega,f}$ is the probability measure on the surface of the spheres $E_{\Omega,f} = \{\boldsymbol{\mu} : \boldsymbol{\mu}^t\Omega\boldsymbol{\mu} = f^2\}$ induced by the probability measure $\mathcal{B}$ for polar coordinates on the unit sphere (Folland, 1999, Chap. 2), normed so that $\sigma_{\Omega,f}(E_{\Sigma,f}) = 1$. Let us derive the prior $g(\boldsymbol{\beta}_2)$ induced by the uniform distribution on $\boldsymbol{\mu}$:

$$\begin{aligned} g(\boldsymbol{\beta}_2) &= \int_{E_{\Omega,f}} f_t(\boldsymbol{\beta}_2 \mid \nu, \boldsymbol{\mu}, r^2\Omega^{-1})\, \mathrm{d}\sigma_{\Omega,f}(\boldsymbol{\mu}) \\ &= \int \frac{\Gamma((\nu+q)/2)}{(\pi\nu)^{q/2}\Gamma(\nu/2)} (r^2)^{-\frac{q}{2}} |\Omega|^{\frac{1}{2}} \left(1 + \frac{1}{\nu r^2}(\boldsymbol{\beta}_2 - \boldsymbol{\mu})^t\Omega(\boldsymbol{\beta}_2 - \boldsymbol{\mu})\right)^{-(\nu+q)/2} \mathrm{d}\sigma_{\Omega,f}(\boldsymbol{\mu}). \end{aligned}$$

Let $\lambda = \sqrt{\boldsymbol{\beta}_2^t\Omega\boldsymbol{\beta}_2}$ and note that

$$(\boldsymbol{\beta}_2 - \boldsymbol{\mu})^t\Omega(\boldsymbol{\beta}_2 - \boldsymbol{\mu}) = \lambda^2 + f^2 - 2\lambda f \frac{\boldsymbol{\beta}_2^t}{\lambda}\Omega\frac{\boldsymbol{\mu}}{f}.$$

By a similar argument as in the last subsection, $\phi = (\frac{1}{\lambda}\boldsymbol{\beta}_2^t)\Omega(\frac{1}{f}\boldsymbol{\mu})$ can be seen to follow a distribution with density given by $f(\phi)$ in Equation A2.

Hence,

$$\begin{aligned} g(\boldsymbol{\beta}_2) &= \int_{-1}^{1} \frac{\Gamma((\nu+q)/2)}{(\pi\nu)^{q/2}\Gamma(\nu/2)}(r^2)^{-\frac{q}{2}}|\Omega|^{\frac{1}{2}}\left(1+\frac{1}{\nu r^2}(\lambda^2+f^2-2\lambda f\phi)\right)^{-(\nu+q)/2} \\ &\qquad \frac{\Gamma(q/2)}{\sqrt{\pi}\Gamma((q-1)/2)}(1-\phi^2)^{(q-1)/2-1}\,\mathrm{d}\phi \\ &= \frac{\Gamma((\nu+q)/2)(\nu r^2)^{\nu/2}}{\pi^{q/2}\Gamma(\nu/2)}|\Omega|^{\frac{1}{2}}(\lambda^2+f^2+\nu r^2)^{-\frac{\nu+q}{2}} \\ &\qquad {}_2F_1\left(\frac{\nu+q}{4};\frac{1}{4}(2+\nu+q);\frac{q}{2};\frac{4f^2\lambda^2}{(\lambda^2+f^2+\nu r^2)^2}\right), \end{aligned}$$

using the hypergeometric function ${}_2F_1$.

It follows that

$$l_1(\boldsymbol{y}\mid\mathcal{M}_0)=\int\left(\iint f(\boldsymbol{y}\mid\boldsymbol{\beta}_1,\boldsymbol{\beta}_2,\epsilon)f(\epsilon)\,\mathrm{d}\boldsymbol{\beta}_1\,\mathrm{d}\epsilon\right)g(\boldsymbol{\beta}_2)\,\mathrm{d}\boldsymbol{\beta}_2.$$

We now move to standardized effects by a change of variable: $\boldsymbol{\gamma}=\Omega^{\frac{1}{2}}\boldsymbol{\beta}_2$. Note that $\lambda^2=\boldsymbol{\gamma}^t\boldsymbol{\gamma}$ and that

$$l_1(\boldsymbol{y}\mid\mathcal{M}_0)=\int\left(\iint f(\boldsymbol{y}\mid\boldsymbol{\beta}_1,\Omega^{-\frac{1}{2}}\boldsymbol{\gamma},\epsilon)f(\epsilon)\,\mathrm{d}\boldsymbol{\beta}_1\,\mathrm{d}\epsilon\right)h(\boldsymbol{\gamma})\,\mathrm{d}\boldsymbol{\gamma}.$$

with

$$\begin{aligned} h(\boldsymbol{\gamma}) &= \frac{\Gamma((\nu+q)/2)(\nu r^2)^{\nu/2}}{\pi^{q/2}\Gamma(\nu/2)}(\lambda^2+f^2+\nu r^2)^{-\frac{\nu+q}{2}} \\ &\qquad {}_2F_1\left(\frac{\nu+q}{4};\frac{1}{4}(2+\nu+q);\frac{q}{2};\frac{4f^2\lambda^2}{(\lambda^2+f^2+\nu r^2)^2}\right). \end{aligned}$$

In polar coordinates, $\boldsymbol{\gamma}$ is decomposed into its norm λ and its angle as indicated by its directional vector of norm 1: $\boldsymbol{\gamma}/\lambda\in E$, where E is as before the unit sphere. We can therefore split the integration over the distribution of $\boldsymbol{\gamma}$ into an outer integration over the distribution of the norm of $\boldsymbol{\gamma}$ with density $m(\lambda)$ (to be derived in the sequel) and an inner integral over its angle. Setting $\boldsymbol{\gamma}=\lambda\boldsymbol{\gamma}'$, it follows from Theorem 2.49 in Folland (1999) that

$$l_1(\boldsymbol{y}\mid\mathcal{M}_0)=\int_0^\infty\int_{E_{I,1}}\left(\iint f(\boldsymbol{y}\mid\boldsymbol{\beta}_1,\Omega^{-\frac{1}{2}}\boldsymbol{\gamma},\epsilon)f(\epsilon)\,\mathrm{d}\boldsymbol{\beta}_1\,\mathrm{d}\epsilon\right)h(\boldsymbol{\gamma})\,\mathrm{d}(B\mathcal{B})(\boldsymbol{\gamma}')\lambda^{q-1}\,\mathrm{d}\lambda,$$

where $B=\frac{2\pi^{q/2}}{\Gamma(q/2)}$ is the measure (area or volume) of the unit sphere. The function $h(\boldsymbol{\gamma})$ depends on $\boldsymbol{\gamma}$ only via its norm $\lambda=||\boldsymbol{\gamma}||$ and thus, we can

define $h'(\lambda)$ as $h(\boldsymbol{\gamma})$. It follows that

$$
\begin{aligned}
l_1(\boldsymbol{y} \mid \mathcal{M}_0) &= \int_0^\infty \int_{E_{I,1}} \left(\iint f(\boldsymbol{y} \mid \boldsymbol{\beta}_1, \Omega^{-\frac{1}{2}}\boldsymbol{\gamma}, \epsilon) f(\epsilon)\, \mathrm{d}\boldsymbol{\beta}_1\, \mathrm{d}\epsilon \right) \mathrm{d}\mathcal{B}(\boldsymbol{\gamma}') \\
& \qquad B\lambda^{q-1} h'(\lambda)\, \mathrm{d}\lambda.
\end{aligned}
$$

Setting $m(\lambda) = B\lambda^{q-1}h'(\lambda)$, it follows from Corollary 2.51 in Folland (1999) that

$$
1 = \int h(\boldsymbol{\gamma})\, \mathrm{d}\boldsymbol{\gamma} = \int_0^\infty m(\lambda)\, \mathrm{d}\lambda,
$$

so that $m(\lambda)$ is seen to be a density. It follows that

$$
\begin{aligned}
m(\lambda) = 2\frac{\Gamma((\nu+q)/2)}{\Gamma(\nu/2)\Gamma(q/2)} (\nu r^2)^{\nu/2} \lambda^{q-1} (\lambda^2 + f^2 + \nu r^2)^{-\frac{\nu+q}{2}} \\
{}_2F_1\left(\frac{\nu+q}{4}; \frac{1}{4}(2+\nu+q); \frac{q}{2}; \frac{4f^2\lambda^2}{(\lambda^2+f^2+\nu r^2)^2} \right).
\end{aligned}
$$

It follows that the density of the effect size $\lambda^2 = \boldsymbol{\beta}_2^t \Sigma/(N-p)\boldsymbol{\beta}_2 = \boldsymbol{\gamma}^t\boldsymbol{\gamma}$ induced by the priors is given by

$$
\begin{aligned}
qe(\lambda^2) = \frac{\Gamma((\nu+q)/2)}{\Gamma(\nu/2)\Gamma(q/2)} (\nu r^2)^{\frac{\nu}{2}} (\lambda^2)^{\frac{q}{2}-1} (\lambda^2 + f^2 + \nu r^2)^{-\frac{\nu+q}{2}} \\
{}_2F_1\left(\frac{\nu+q}{4}; \frac{1}{4}(2+\nu+q); \frac{q}{2}; \frac{4f^2\lambda^2}{(\lambda^2+f^2+\nu r^2)^2} \right).
\end{aligned}
$$

It follows that the Bayes factor for effect-size priors is given by

$$
\begin{aligned}
BF_{10} &= \frac{l(\boldsymbol{y} \mid \mathcal{M}_1)}{l(\boldsymbol{y} \mid \mathcal{M}_0)} \\
&= \int \frac{f_F(F \mid q, M-q, M\lambda^2)}{f_F(F \mid q, M-q, 0)} qe(\lambda^2)\, \mathrm{d}\lambda^2.
\end{aligned}
$$

A.2.3 Prior Induced by Moment Priors on the Effect-Size Parameter

The moment prior for $\boldsymbol{\beta}^2$ focused on effect size λ^2 is given by ($\xi = \frac{(\nu+q-2)f^2}{2\nu}$)

$$
\frac{2(\nu-2)}{q(\nu+q-2)f^2} \boldsymbol{\beta}_2^t \Omega \boldsymbol{\beta}_2 f_t(\boldsymbol{\beta}_2 \mid \nu, \mathbf{0}, \xi\Omega^{-1}).
$$

Moving to standardized effects $\boldsymbol{\gamma} = \Omega^{\frac{1}{2}}\boldsymbol{\beta}_2$, the prior on $\boldsymbol{\gamma}$, $h(\boldsymbol{\gamma})$ is seen to be

$$
h(\boldsymbol{\gamma}) = \xi^{-\frac{q}{2}} \frac{2(\nu-2)}{q(\nu+q-2)f^2} \boldsymbol{\gamma}^t\boldsymbol{\gamma} \frac{\Gamma(\frac{\nu+q}{2})}{(\pi\nu)^{q/2}\Gamma(\nu/2)} \left(1 + (\nu\xi)^{-1}\boldsymbol{\gamma}^t\boldsymbol{\gamma}\right)^{-\frac{\nu+q}{2}}.
$$

The function $h(\boldsymbol{\gamma})$ depends on $\boldsymbol{\gamma}$ only via its norm $\lambda = ||\boldsymbol{\gamma}||$ and thus, we can define $h'(\lambda)$ as $h(\boldsymbol{\gamma})$. Following the same steps as in the previous subsection, it follows that the prior density for λ is given by

$$\begin{aligned} m(\lambda) &= \frac{2\pi^{q/2}}{\Gamma(q/2)}\lambda^{q-1}h'(\lambda) \\ &= \frac{2\Gamma(\frac{\nu+q}{2})}{\Gamma(q/2)\Gamma(\nu/2)}\frac{2(\nu-2)}{q(\nu+q-2)f^2}(\nu\xi)^{-\frac{q}{2}}\lambda^{q+1}\left(1+(\nu\xi)^{-1}\lambda^2\right)^{-\frac{\nu+q}{2}}. \end{aligned}$$

Hence, the density induced on λ^2 is

$$qm(\lambda^2) = \frac{\Gamma(\frac{\nu+q}{2})}{\Gamma(q/2)\Gamma(\nu/2)}\frac{2(\nu-2)}{q(\nu+q-2)f^2}((\xi\nu)^{-1}\lambda^2)^{q/2}\left(1+(\xi\nu)^{-1}\lambda^2\right)^{-\frac{\nu+q}{2}}.$$

It follows that the Bayes factor for moment priors is given by

$$\begin{aligned} BF_{10} &= \frac{l(\boldsymbol{y} \mid \mathcal{M}_1)}{l(\boldsymbol{y} \mid \mathcal{M}_0)} \\ &= \int \frac{f_F(F \mid q, M-q, M\lambda^2)}{f_F(F \mid q, M-q, 0)} qm(\lambda^2)\, \mathrm{d}\lambda^2. \end{aligned}$$

It is also not difficult to show that the same formulae result for $q = 1$.

A.3 Coding ANOVA Models as Regression Models

Framing ANOVA analyses as equivalent regression model analyses, the first predictor $\boldsymbol{x}_1 = (1, \ldots, 1)$ of the full model codes an intercept, whereas the other predictors code the factors and interactions using, for example, sum-to-zero contrasts (e.g. Judd et al., 2017). Each main effect and interaction is thereby coded by a subset of predictors, and the F tests in ANOVA contrast a reduced model from which certain effects and/or interactions are omitted against a full model that includes these effects and/or interactions.

There are different ways to code designs that include within-participant factors for equivalent regression analyses. The conceptually easiest way to do this is through the use of orthonormal contrasts of the data (Lane, 2016). This involves transforming the data using orthonormal contrasts such as polynomial contrasts for each within-participant factor with more than two levels (for factors with two levels, difference scores can be used). For example, in a design with one within-participant factor with three levels, let the data for the $s-$th person in the $i-$th condition be y_{si}. We then compute two scores for each person, one for the linear trend $l_{s1} = -\frac{\sqrt{2}}{2}y_{s_1} + \frac{\sqrt{2}}{2}y_{s3}$ and one for the

quadratic trend $l_{s2} = -\frac{1}{\sqrt{6}}y_{s1} + \frac{2}{\sqrt{6}}y_{s2} - \frac{1}{\sqrt{6}}y_{s3}$. If the sphericity assumption is met, the variance-covariance matrix for $(l_{s1}, l_{s2})^t$ is diagonal with equal values in the diagonal. In other words, we can treat the transformed data as if they stemmed from a between-participants design. Note that we have lost S data points with S being the number of participants so that the transformed data comprise $S \times (3 - 1)$ data points. The full regression model uses $q = 3 - 1$ parameters for the effects of the within factor, the reduced model is the trivial one with $\hat{l}_{si} = 0$ and $p = 0$ – an intercept is not needed because we are dealing with data contrasts with expected value equal to zero. Note that sphericity is not an extraneous assumption that needs to be added to the distributional assumptions for regression analyses as stated in the body of the paper. Instead, if the full regression model holds, sphericity is implied for the untransformed data (Lane, 2016).

For designs with several within factors, see Lane (2016) on how to define interaction-specific contrasts for a test of their interactions. For tests of effects involving only a subset of the within factors in the design, we first aggregate the data across the levels of the within factors not in the effect under scrutiny (Maxwell & Delaney, 2004, Chap. 12) before moving to orthonormally transformed data. Between-participants factors can be seamlessly integrated into these models for tests involving interactions of within and between factors – after moving to the transformed data, there is no longer any principled difference between within and between factors. Tests involving only between factors can simply be done on the data aggregated across the within conditions.

Another way to code ANOVA designs with within-participant factors proceeds from the untransformed data and introduces parameters for persons and their interactions with within factors (excepting the highest order interaction of persons and within factors; Rouder et al., 2012) in defining reduced and full regression models for testing specific effects and interactions involving such factors. This approach also involves collapsing the data across within factors not involved in the effect or interaction to be tested. The approach leads to the same F values and degrees of freedom as the approach just considered and to the same effect-size and moment Bayes factors. None of these coding schemes need concern the user of our `R` package. All that is required for computing the new Bayes factors for an effect or interaction is its F value from the univariate repeated-measures ANOVA or, as the case may be, mixed ANOVA and its degrees of freedom.

A complication regarding the interpretation of effect size f in designs with within-participant factors should be noted. The effect-size parameters f

implied by these analyses differ from the ones obtained if the same hypothesis tests are conducted in a between-participants setup with different groups of participants realizing the levels of the within-participant factor. Consider, for example, the test for a main effect of a between-participants factor in a design with a within-participant factor A. The test can proceed on the personwise means of the data across the levels of the factor A. If A has m levels, it is easy to show that the variance of these means is $\sigma_b^2 = u\sigma^2$ with $u = \frac{1}{m}(1 + (m-1)\rho)$, where ρ is the common correlation between the repeated measures (note that we assume compound symmetry at this point for the sake of simplicity) and thus, in the definitions of the non-centrality parameter and of f, we would divide by σ_b^2 instead of by σ^2. If we want to define f in terms of σ instead of σ_b, f should be divided by $\sqrt{u}$. Similar correction factors apply to tests of effects involving within factors (see Faul et al., 2007, Table 3, and see Lakens, 2013, for more discussion of this issue).

A.4 Framing Invariance

To show framing invariance, we have to show how the one-sample and two-sample t test can be framed as comparisons of a reduced and a full linear model as per the regression Bayes factor. For the one-sample t test, the reduced model has zero predictors and states $\hat{\boldsymbol{y}} = X_1\boldsymbol{\beta}_1 = \mathbf{0}$. Hence, $p = 0$. The full model has one predictor, the intercept, with $q = 1$ so that $X_2 = \mathbf{1}_N$, where $\mathbf{1}_N$ is a vector of length N with cell entries 1. From here, it is easy to see that the assumptions from Table 6 in the body of the paper for the regression case are the same as those from Table 3 in the body of the paper. This means that the same distributions are specified in both cases, yielding the same Bayes factor.

For the two-sample case, the reduced model has one predictor, the intercept, $X_1 = \mathbf{1}_N$, hence $p = 1$, whereas the full model has the additional predictor $X_2 = \frac{1}{2}(-\mathbf{1}_{N_1}^t, \mathbf{1}_{N_2}^t)^t$, hence $q = 1$. The matrix Σ is then given by $X_2^t(I - \frac{1}{N}\mathbf{1}_{N\times N})X_2$, where $\mathbf{1}_{N\times N}$ is the N by N matrix with cell entries 1. Hence, $\Sigma = N_1N_2/N$ and $\Sigma/(N-p) = \Sigma/(N-1) = M/(N-1)$, where $M = N_1N_2/N$. From here, framing invariance follows when the relations between d_e and f (and the scaling factors r for the regression model and the two-sample t test with effect-size priors) stated in the body of the paper are taken into account.

A.5 Large-Sample Consistency for Moment Bayes Factors

Because of *framing invariance*, it is sufficient to show large-sample consistency for the moment Bayes factor for regression models. The proofs showing consistency of the Bayes factor for models with fixed dimensionality do not immediately work for priors such as the moment priors with value zero at the true parameter value. The reason is that asymptotic expansions of the logarithm of the Bayes factor $\log(BF_{10})$ are additively contributed to by $\log(f_1(\hat{\boldsymbol{\theta}}_1)/f_0(\hat{\boldsymbol{\theta}}_0))$ involving the ratio of the prior densities of the two models at the respective maximum likelihood estimates (Chib & Kuffner, 2016), which is normally bounded in probability as N becomes large and can therefore be ignored in asymptotic treatments of Bayes factor consistency. If the reduced model is true, the maximum likelihood estimates converge to the true parameter values, which in the case of the full model means that $f_1(\hat{\boldsymbol{\theta}}_1)$ will, however, tend to zero as N tends to infinity so that the above term is no longer bounded. Fortunately, $\log(f_1(\hat{\boldsymbol{\theta}}_1)/f_0(\hat{\boldsymbol{\theta}}_0))$ is thereby seen to tend to minus infinity as N becomes large so that the additive term will in fact additionally speed up the convergence of the Bayes factor to zero if the reduced model is true (see Johnson & Rossell, 2010, for a more technical explanation). No such problem arises if the reduced model is false, because the maximum-likelihood estimate will converge to a value at which the prior is positive.

A.6 Consistency in Information

Because of *framing invariance*, it is sufficient to show consistency in information for the Bayes factor for regression models as $F \to \infty$ or, equivalently, $R^2 = R^2_{\text{partial}} \to 1$.In terms of R^2, the effect-size Bayes factor is given by (see Equation A3)

$$B_{10}(R^2) = \int_0^\infty e^{-\frac{1}{2}M\lambda^2}\, {}_1F_1(\frac{M}{2}; \frac{q}{2}; \frac{1}{2}M\lambda^2 R^2) qe(\lambda^2)\, \mathrm{d}\lambda^2.$$

Because the term in qe involving ${}_2F_1$ is greater or equal to 1, there is a constant $P_1 > 0$ so that

$$B_{10}(R^2) \geq P_1 \int_0^\infty e^{-\frac{1}{2}M\lambda^2}\, {}_1F_1(\frac{M}{2}; \frac{q}{2}; \frac{1}{2}M\lambda^2 R^2)(\lambda^2)^{\frac{q}{2}-1}(\lambda^2+f^2+\nu r^2)^{-\frac{\nu+q}{2}}\, \mathrm{d}\lambda^2.$$

Because ${}_1F_1(a;b;c)$ tends to $\frac{\Gamma(a)}{\Gamma(b)} e^z z^{a-b}$ as z becomes large, there are constants $P_2 > 0$ and $P_3 > 0$ so that

$$B_{10}(R^2) \geq P_2 \int_{P_3}^\infty e^{-\frac{1}{2}M\lambda^2(1-R^2)}(\frac{1}{2}MR^2)^{\frac{M-q}{2}}(\lambda^2)^{\frac{M-q}{2}}(\lambda^2)^{\frac{q}{2}-1}(\lambda^2+f^2+\nu r^2)^{-\frac{\nu+q}{2}}\, \mathrm{d}\lambda^2.$$

Hence, if $R^2 \geq \frac{1}{2}$ and noting that $\frac{\lambda^2}{\lambda^2+f^2+\nu r^2} \to 1$ as λ becomes large, there are constants $P_4 > 0$ and $P_5 > 0$ so that

$$B_{10}(R^2) \geq P_4 \int_{P_5}^{\infty} e^{-\frac{1}{2}M\lambda^2(1-R^2)}(\lambda^2)^{\frac{M-q-\nu}{2}-1}\,\mathrm{d}\lambda^2.$$

Setting $Q = (\frac{1}{2}M(1-R^2))^{-1/2}$, it follows that $Q \to \infty$ as $R^2 \to 1$ and that (for $Q \geq P_5$)

$$B_{10}(R^2) \geq P_4 \int_{P_5}^{Q} e^{-\frac{1}{2}MQ^2(1-R^2)}(\lambda^2)^{\frac{M-q-\nu}{2}-1}\,\mathrm{d}\lambda^2 = P_4 e^{-1}\int_{P_5}^{Q}(\lambda^2)^{\frac{M-q-\nu}{2}-1}\,\mathrm{d}\lambda^2.$$

The latter integral is easily seen to tend to infinity as $Q \to \infty$ if $M-q-\nu \geq 0$, that is if $N \geq p+q+\nu$.

A very similar derivation shows that the moment Bayes factor tends to infinity as $R^2 \to 1$ for $N \geq p+q+\nu-2$.

A.7 Predictive Matching

Because of framing invariance, it is sufficient to show predictive matching for the regression model. We begin with the case that we have $N = p+q$ observations with a design matrix X of full rank $p+q$. Note that in this case $R^2_{\text{partial}} = 1$, because $\hat{\boldsymbol{y}} = \boldsymbol{y}$. Hence, with $q = qm$ or $q = qe$, the Bayes factor is

$$B_{10}(R^2_{\text{partial}} = 1) = \int_0^{\infty} e^{-\frac{1}{2}M\lambda^2}\,{}_1F_1(\frac{M}{2};\frac{q}{2};\frac{1}{2}M\lambda^2)q(\lambda^2)\,\mathrm{d}\lambda^2.$$

Because $M-q = N-p-q = 0$ and $e^{-z}\,{}_1F_1(a;b;z) = {}_1F_1(b-a,b,-z)$ for all a, b, and z, this further simplifies to:

$$B_{10}(1) = \int_0^{\infty} {}_1F_1(0;\frac{q}{2};-\frac{1}{2}M\lambda^2)q(\lambda^2)\,\mathrm{d}\lambda^2.$$

Because ${}_1F_1(0,b,-z) = 1$ for all b and z, we have

$$B_{10} = \int_0^{\infty} q(\lambda^2)\,\mathrm{d}\lambda^2 = 1.$$

If $N < p+q$, the matrix X_2 is of rank $N-p = q' < q$. Some of the β_2 parameters are therefore redundant and we can reparametrize the model using fewer additional parameters $\boldsymbol{\beta}_2'$ (i.e., q') parameters in the full model and a design matrix $X = (X_1, X_2')$ so that X' is a $(p+q') \times (p+q')$ matrix of full

rank (because the priors for $\boldsymbol{\beta}_2$ are proper priors, the redundant parameters that do no longer occur in the reparameterized data likelihood can be integrated out in computing the marginal likelihood of the full model). As just shown, this yields $B_{10} = 1$.

The second case of uninformative data is given if the design matrix X_1 for the reduced model has rank p, but the design matrix for the full model (X_1, X_2) has also rank p. In this case, a linear transformation of the parameters $\boldsymbol{\beta}_2$ exists so that $X_1(\boldsymbol{\beta}_1 + L\sigma\boldsymbol{\beta}_2) = X_1\boldsymbol{\beta}_1 + \sigma X_2\boldsymbol{\beta}_2$ for all $\boldsymbol{\beta}_2$ with a $p \times q$ matrix L. If $f(\boldsymbol{\beta}_1)$ and $f(\boldsymbol{\beta}_2, \sigma)$ are the priors on the model parameters (with $f(\boldsymbol{\beta}_1) = 1$),

$$l(\mathbf{y} \mid \mathcal{M}_{\mathbf{1}}) = \iint \left(\int \mathbf{f}(\mathbf{y} \mid \boldsymbol{\beta}_{\mathbf{1}}, \boldsymbol{\beta}_{\mathbf{2}}, \sigma)\mathbf{f}(\boldsymbol{\beta}_{\mathbf{1}})\,\mathrm{d}\boldsymbol{\beta}_{\mathbf{1}} \right) \mathbf{f}(\boldsymbol{\beta}_{\mathbf{2}}, \sigma)\,\mathrm{d}\boldsymbol{\beta}_{\mathbf{2}}\,\mathrm{d}\sigma.$$

Make a change of variable in the inner integral from $\boldsymbol{\beta}_1$ to $\boldsymbol{\beta}_1^* = \boldsymbol{\beta}_1 + L\sigma\boldsymbol{\beta}_2$. Because the Jacoby factor for this change of variable is 1, and $X_1(\boldsymbol{\beta}_1 + L\sigma\boldsymbol{\beta}_2) = X_1\boldsymbol{\beta}_1^*$, the value of the inner integral with the new integration variable $\boldsymbol{\beta}_1^*$ is seen to be (a) not a function of $\boldsymbol{\beta}_2$ and (b) identical to the analogous integral computed under $\mathcal{M}_0$. From here, it is easy to see that again $BF_{10} = 1$.

A.8 Directional Tests, Tests against Values Other than Zero, Tests of Interval Hypotheses

Directional tests such as $H_1 : \theta \geq 0$ can be easily accommodated by restricting the integration in the computation of the Bayes factors (see Table 4) to range from 0 to ∞ instead of from $-\infty$ to ∞. Tests against values θ_0 other than zero (i.e., of $H_0 : \theta = \theta_0$) can be accommodated by data transformations and then testing against zero. For one-sample and two-sample t tests, this amounts to conducting the test against zero on transformed data $\tilde{y}_i = y_i - \theta_0$. For regression problems ($H_0 : \boldsymbol{\beta}_2 = \boldsymbol{\theta}_0$), the data should be transformed according to $\tilde{\boldsymbol{y}} = \boldsymbol{y} - X_2\boldsymbol{\theta}_0$.

Interval hypotheses specified in terms of effects d, for example $H_0 : |d| \leq \epsilon$ versus $H_1 : |d| > \epsilon$ can be tested in a two-step procedure for one-sample and two-sample t tests. Restricting the integration in Table 4 to range from $-d$ to d yields a Bayes factor B_1 testing the interval hypothesis against the strict null; restricting the integation to values outside the interval yields a Bayes factor B_2 testing the above H_1 against the strict null. The Bayes factor for testing the above H_1 against H_0 is then obtained as the quotient ${}^{B_2}/_{B_1}$. For regression/ANOVA models, tests of interval hypotheses such as $H_0 : f^2 \leq \epsilon$ versus $H_1 : f^2 > \epsilon$ proceed analogously.

A.9 Bayes Factors Based on p values versus Bayes Factors Based on the Raw Data

The test statistics in significance tests are constructed so as to satisfy two desiderata: Their distribution should depend only on the parameter(s) θ contrasted in the hypothesis test and not on other model parameters ρ shared by the underlying models (e.g., Johnson, 2005).[2] Furthermore, they should ideally capture all of the information that the data contain about the critical parameter(s). This latter desideratum is perfectly satisfied if the test statistic T is a sufficient statistic for θ, meaning that the distribution of the data given T does not depend upon θ (Halmos & Savage, 1949). If so, we can factorize the likelihood of the data $\boldsymbol{y}$ as follows:

$$f(\boldsymbol{y} \mid \rho, \theta) = f(\boldsymbol{y} \mid \rho, T(\boldsymbol{y}))f(T(\boldsymbol{y}) \mid \theta). \tag{A5}$$

From here, it is easy to see that the ratio of the marginal likelihoods of the data under $\mathcal{M}_1$ and $\mathcal{M}_0$, that is the Bayes factor based on the original data, reduces to the Bayes factor based on p-values.

For many significance tests, including the t and F tests considered here, the test statistic T does not reach the ideal case of providing a sufficient statistic, but it is nevertheless constructed so that its distribution depends only on the critical parameters while capturing as much information about them as possible under this constraint. This suggests that the Bayes factor based directly on the value t of the test statistic T as per Equation 5 in the body of the paper will in general make appropriate use of the information that the data contain on the hypothesis test in question. Rouder and colleagues' furthermore argue (Rouder & Morey, 2012; Rouder et al., 2009, 2012) that the Bayes factor does not depend strongly on the distributional assumptions made for parameters outside the hypotheses that are contrasted and that are shared by both models (see also O'Hagan & Forster, 2004, p. 179, for a similar argument). This intuition is supported by Du et al.'s (2019) sensitivity analyses on the Bayesian t test regarding the effects of different priors on the variance parameter σ^2 as long as relatively vague priors are chosen for σ^2. This and the equivalence of the p value-based Bayes factor and the default, effect-size, and moment Bayes factors suggest that the p value-based Bayes factor will in general be similar to a Bayes factor computed from a full set of distributional assumptions on all model parameters when vague priors are used for the parameters shared by both models.

[2]This is often true only after a reparameterization using effect-size parameters

A.10 Applications: Values of the Fractional and Adjusted Fractional Bayes Factors

A.10.1 t Tests

Table A1 shows the fractional and adjusted fractional Bayes factor for the t test examples shown in Table 7 in the body of the paper.

Table A1: Default, Fractional and Adjusted Fractional Bayes Factors for t Tests

$t(79)$	Default BF	Fractional BF	Adjusted Fractional BF
2.03	0.64	0.65	0.69
2.24	0.98	0.99	1.05

Note. BF = Bayes factor.

A.10.2 Regression Models

Considering the regression example from the body of the paper, the fractional and adjusted fractional Bayes factors revealed decisive evidence against the null model for all non-null models like the default, effect-size and moment Bayes factors. Furthermore, this was also true for the comparison of reduced models relative to the full model for all but three reduced models.

Table A2 shows the values of the fractional and adjusted fractional Bayes factors for the three reduced models in the same format as in Table 8 in the body of the paper.

Table A2: Default, Fractional and Adjusted Fractional Bayes Factors for Reduced Models against the Full Models

	Model	R_p^2	Default BF	Fractional BF	Adjusted Fractional BF
$\mathcal{M}_2$	L+G+D	.0126	4.41	5.57	5.50
$\mathcal{M}_4$	G+P+D	.0002	12.97	16.27	16.27
$\mathcal{M}_9$	G+D	.0136	61.03	53.23	52.15

Note. R_p^2 = partial R^2; BF = Bayes factor; L = local climate variation; G = global temperature; P = parasite load; D = population density. R_p^2 values are computed from the different models' R^2 values reported by Rouder and Morey (2012). Since these are rounded to four digits after the decimal point, results may differ slightly if the original data would have been available for analysis.

A.10.3 ANOVA

Table A3 shows the values of the fractional and adjusted fractional Bayes factors for the ANOVA problem analyzed in the body of the paper (see Figure 6 in the body of the paper).

Table A3: Default, Fractional and Adjusted Fractional Bayes Factors for ANOVA Example

Effect	Default BF	Fractional BF	Adjusted Fractional BF
Orientation	141.21	18.46	24.46
Frequency	0.48	0.14	0.14
Interaction	0.50	0.15	0.18

Note. BF = Bayes factor.

References

Abramowitz, M., & Stegun, I. A. (1972). *Handbook of mathematical functions with formulas, graphs, and mathematical tables.* U.S. Government Printing Office.

Chib, S., & Kuffner, T. A. (2016). *Bayes factor consistency.* ArXiv. https://doi.org/10.48550/arXiv.1607.00292

Du, H., Edwards, M. C., & Zhang, Z. (2019). Bayes factor in one-sample tests of means with a sensitivity analysis: A discussion of separate prior distributions. *Behavior Research Methods*, *51*, 1998–2021. https://doi.org/10.3758/s13428-019-01262-w

Faul, F., Erdfelder, E., Lang, A.-G., & Buchner, A. (2007). G*Power 3: A flexible statistical power analysis program for the social, behavioral, and biomedical sciences. *Behavior Research Methods*, *39*, 175–191. https://doi.org/10.3758/BF03193146

Folland, G. B. (1999). *Real analysis: Modern techniques and their applications* (2nd ed.). John Wiley & Sons.

Gronau, Q. F., Ly, A., & Wagenmakers, E.-J. (2020). Informed Bayesian *t*-tests. *The American Statistician*, *74*, 137–143. https://doi.org/10.1080/00031305.2018.1562983

Halmos, P. R., & Savage, L. J. (1949). Application of the Radon-Nikodym theorem to the theory of sufficient statistics. *The Annals of Mathematical Statistics*, *20*, 225–241. https://doi.org/10.1214/aoms/1177730032

Johnson, V. E. (2005). Bayes factors based on test statistics. *Journal of the Royal Statistical Society Series B: Statistical Methodology*, *67*, 689–701.

Judd, C. M., McClelland, G. H., & Ryan, C. S. (2017). *Data analysis: A model comparison approach to regression, ANOVA, and beyond* (3rd ed.). Routledge.

Lakens, D. (2013). Calculating and reporting effect sizes to facilitate cumulative science: A practical primer for t-tests and ANOVAs. *Frontiers in Psychology*, *4*. https://doi.org/10.3389/fpsyg.2013.00863

Lane, D. M. (2016). The assumption of sphericity in repeated-measures designs: What it means and what to do when it is violated. *The Quantitative Methods for Psychology*, *12*, 114–122. https://doi.org/10.20982/tqmp.12.2.p114

Maxwell, S. E., & Delaney, H. D. (2004). *Designing experiments and analyzing data: A model comparison perspective* (2nd ed.). Lawrence Erlbaum Associates Publishers.

O'Hagan, A., & Forster, J. J. (2004). *Kendall's advanced theory of statistics: Bayesian inference* (2nd ed., Vol. 2B). Arnold.

Rouder, J. N., & Morey, R. D. (2012). Default Bayes factors for model selection in regression. *Multivariate Behavioral Research*, *47*, 877–903. https://doi.org/10.1080/00273171.2012.734737

Rouder, J. N., Morey, R. D., Speckman, P. L., & Province, J. M. (2012). Default Bayes factors for ANOVA designs. *Journal of Mathematical Psychology*, *56*, 356–374. https://doi.org/10.1016/j.jmp.2012.08.001

Rouder, J. N., Speckman, P. L., Sun, D., Morey, R. D., & Iverson, G. (2009). Bayesian t tests for accepting and rejecting the null hypothesis. *Psychonomic Bulletin & Review*, *16*, 225–237. https://doi.org/10.3758/PBR.16.2.225
